# Identification of genes expressed in a mesenchymal subset regulating prostate organogenesis using tissue and single cell transcriptomics

**DOI:** 10.1038/s41598-017-16685-8

**Published:** 2017-11-27

**Authors:** Nadia Boufaied, Claire Nash, Annie Rochette, Anthony Smith, Brigid Orr, O. Cathal Grace, Yu Chang Wang, Dunarel Badescu, Jiannis Ragoussis, Axel A. Thomson

**Affiliations:** 1 Department of Surgery, Division of Urology, Cancer Research Program, McGill University Health Centre, 1001 Decarie Boulevard, Montreal, Quebec, H4A 3J1 Canada; 2 MRC Human Reproductive Sciences Unit, The Queen’s Medical Research Institute, 47 Little France Crescent, Edinburgh, EH16 4TJ UK; 3 McGill University and Genome Quebec Innovation Centre, 740 Dr. Penfield Avenue, Montreal, H3A 0G1 Canada

## Abstract

Prostate organogenesis involves epithelial growth controlled by inductive signalling from specialised mesenchymal subsets. To identify pathways active in mesenchyme we used tissue and single cell transcriptomics to define mesenchymal subsets and subset-specific transcript expression. We documented transcript expression using Tag-seq and RNA-seq in female rat Ventral Mesenchymal Pad (VMP) as well as adjacent urethra comprised of smooth muscle and peri-urethral mesenchyme. Transcripts enriched in female VMP were identified with Tag-seq of microdissected tissue, RNA-seq of cell populations, and single cells. We identified 400 transcripts as enriched in the VMP using bio-informatic comparisons of Tag-seq and RNA-seq data, and 44 were confirmed by single cell RNA-seq. Cell subset analysis showed that VMP and adjacent mesenchyme were composed of distinct cell types and that each tissue contained two subgroups. Markers for these subgroups were highly subset specific. Thirteen transcripts were validated by qPCR to confirm cell specific expression in microdissected tissues, as well as expression in neonatal prostate. Immunohistochemical staining demonstrated that Ebf3 and Meis2 showed a restricted expression pattern in female VMP and prostate mesenchyme. We conclude that prostate inductive mesenchyme shows limited cellular heterogeneity and that transcriptomic analysis identified new mesenchymal subset transcripts associated with prostate organogenesis.

## Introduction

The development of the prostate is regulated by androgens and mesenchymal:epithelial interactions. Several studies have demonstrated that paracrine acting factors made in the mesenchyme play key roles in regulating male reproductive organogenesis. Pathways such as Fgf, Wnt, TGFbeta, Shh, Notch, and others have been identified as participating in prostate development, though it is uncertain whether our knowledge of regulatory pathways is comprehensive (reviewed in^1^). Transcriptional profiling has been applied to whole prostate organs, both in development and adulthood^2–6^. These studies have identified dynamic expression of many pathways. However, the cellular complexity and proportions of different cell types within organs has led to difficulty in attribution of individual transcripts to defined cell subsets, as well as being confounded by changes in cell proportions over time or following hormonal manipulation. Within these datasets, it is difficult to deconvolute pathways expressed in either stromal or epithelial tissue compartments, although some studies have focussed upon mesenchymal and stromal tissue^7,8^. Since mesenchyme is known to regulate organogenesis as well as mediate the effects of hormones upon development, it is important to identify mesenchymally expressed pathways.

During prostate development, several morphogens are expressed in a subset of mesenchyme termed the Ventral Mesenchymal Pad (VMP) and the peri-urethral mesenchyme. The VMP is most apparent on the ventral aspect of the urethra but it encircles the urethra. Its formation precedes the formation of the ventral, lateral and dorsal prostate lobes. It has been defined as a source of inductive mesenchyme using tissue recombination studies^9^, and several pathways show VMP-specific expression^8,10^. Other regions of the stroma also show subset-specific marker and pathway expression, such as smooth muscle and peri-urethral stroma. A detailed anatomic description of stromal subsets has been described, and defined using specific markers^11–13^. The VMP forms in both males and females^9,14^ and constitutively expresses morphogens such as Fgf10^10^. This has led to the question of whether androgens regulate morphogen expression, which has conflicting experimental support (reviewed in^1,15,16^). It has been shown that androgens control the formation of a sexually dimorphic layer of smooth muscle that separates VMP mesenchyme from nascent prostatic buds^17,18^. This layer may regulate inductive signalling from the VMP, and constitutes part of the hormonal mechanism controlling prostate organogenesis. The smooth muscle hypothesis accounts for the non-dimorphic expression of Fgf10 and other morphogens^16^. A prediction of this hypothesis is that morphogens are constitutively expressed in both males and females but are regulated indirectly by androgens and AR acting in the smooth muscle compartment. We have used VMP isolated from females on the day of birth as our model of prostate mesenchyme, since this is when the tissue is largest and also because female VMP lacks prostatic epithelia and is of low cellular complexity. At the same age in males, the VMP has become the Ventral Prostate, and contains a high proportion of branching epithelia, while the mesenchyme is differentiating into smooth muscle and other fibroblast types. Thus, female VMP is a model system with low cellular complexity that is optimal for identification of molecules involved in prostate development. We have previously used SAGE to identify transcripts specifically expressed in the VMP^8^, and noted that mesenchymal pathways may be dysregulated in cancer-associated fibroblasts, associated with EMT, or neuro-endocrine differentiation of tumours. These studies identified Ptn, Dlk1/Notch2, Scube1, EfnB1/EphB3, and Dcn in prostate development^8,19–22^. One of the limitations of SAGE is its low sensitivity in transcript detection, and next generation RNA sequencing based methods such as Tag-sequencing (Tag-seq) and RNA-sequencing (RNA-seq) have considerably higher sensitivity and superior transcript quantitation, as well as high resolution techniques such as single cell RNAseq.

The rationale for our study was to conduct a high resolution transcriptomic analysis of mesenchymal subsets and to examine homo/heterogeneity in regard to cellular composition, as well as to catalogue transcript expression. Cellular heterogeneity is a significant problem in whole organ and tissue transcriptional profiling. Comparison of transcript profiling from microdissected tissue and single cell RNA-seq (scRNA-seq) was used to identify transcripts with tissue and cell specific expression. The markers and pathways identified by such an approach can be deconvolved in whole organ datasets and prioritised for functional studies. We validated expression of VMP-specific transcripts by qPCR and also confirmed expression in neonatal prostate. Immunohistochemistry of Ebf3 and Meis2 confirmed expression in VMP and prostate mesenchyme.

## Results

### Tag-seq and RNA-seq of microdissected mesenchymal tissues

**Figure 1:**
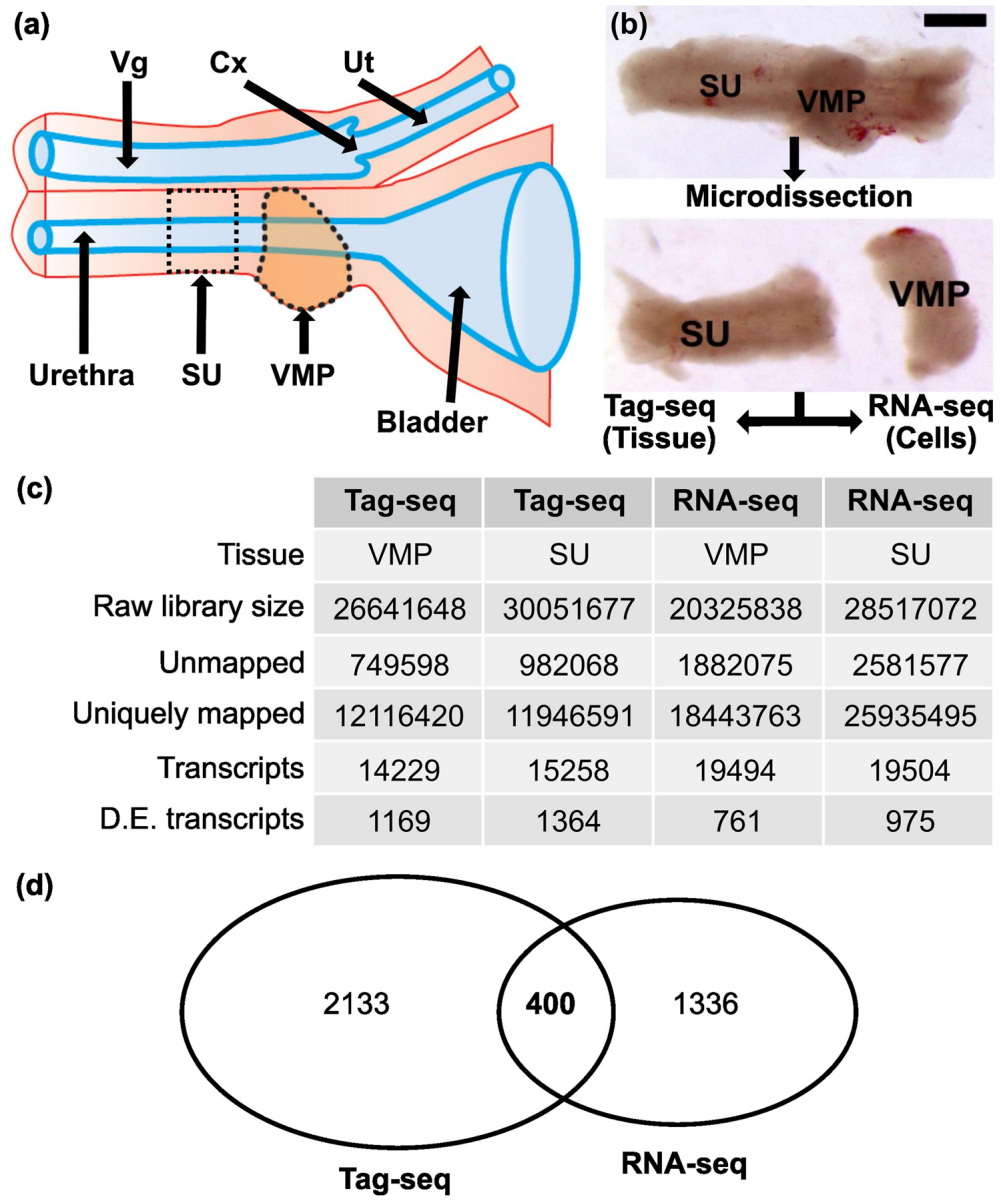
Identification of transcripts differentially expressed in VMP and SU tissues using Tag-seq and RNA-seq. (**a**) A schematic diagram of P0 female urethra illustrating the position of the VMP on the urethra, and an adjacent region of urethra comprised of smooth muscle and urethral epithelia (SU). Vg = Vagina, Cx = Cervix, Ut = Uterus; only one uterine horn is illustrated. (**b**) P0 urethra was micro-dissected to yield VMP mesenchyme and adjacent SU. Micro-dissected VMP consists of pure mesenchyme and lacks epithelia, while SU contains both mesenchyme and epithelia. Tag-seq libraries were prepared from microdissected VMP and SU tissues (Tissue), while RNAseq libraries were prepared from cell suspensions from tissue digested with collagenase (Cells). This enabled the removal of urethral epithelium from the SU cell sample. (**c**) Details of Tag-seq and RNA-seq libraries, and differentially expressed transcripts. A statistical package, NOISeq, was used to define differentially expressed (DE) transcripts in both Tag-seq and RNA-seq datasets. Transcripts with an absolute log2 fold-change (M) ≥ 1.5 and a diverge probability (q) > 0.9 were considered to be differentially expressed. (**d**) Venn diagram showing overlap between DE transcripts in Tag-seq vs RNA-seq data, and identification of 400 DE transcripts common to both techniques.

VMP mesenchyme was microdissected from day of birth (P0) female rat urethra to isolate pure VMP mesenchyme as well as adjacent urethra comprised of smooth muscle, peri-urethral stroma and urethral epithelia (SU). Tissue pools were collected and processed for Tag-seq. As a comparator, pools of microdissected tissues were dissociated using collagenase, and 1000 cells from each pool used for RNA-seq. This dissociation enriched for mesenchymal cells in the SU sample, since epithelia remained intact and were separated from the stromal cells. VMP is wholly mesenchymal, though may contain residual traces of epithelia following dissection. Figure 1a shows a schematic diagram of female urethra, while Fig. 1b shows images of tissue dissection and subsequent analysis. Figure 1c shows Tag-seq and RNA-seq library details, as well as identification of differentially expressed transcripts using NOISeq a method based on empirical distribution suitable for comparison of 2 samples with no replicates^23^. Tag-seq identified 1169 VMP and 1364 SU differentially expressed (DE) transcripts, while RNA-seq identified 761 VMP and 975 SU DE transcripts (Fig. 1c). When transcripts identified as differentially expressed were compared between the two different techniques (Tag-seq and RNA-seq) we observed 400 transcripts as common to both (Fig. 1d). The fold difference of DE transcripts showed similar distributions between Tag-seq and RNA-seq in VMP and SU subsets (Supplementary Figure 1). Comparison of the DE transcripts to the human foetal prostate transcriptome^19,24^ (EMB) co-identified 219 transcripts suggesting that a high proportion (54%) of DE transcripts are expressed during human prostate development (Supplementary Figure 2). At early stages of human prostate development, the organ contains a high proportion of mesenchyme, which likely contributes to the similarity between VMP and foetal prostate transcriptomes.

### Gene Set Enrichment Analysis of subset-specific transcripts

**Figure 2:**
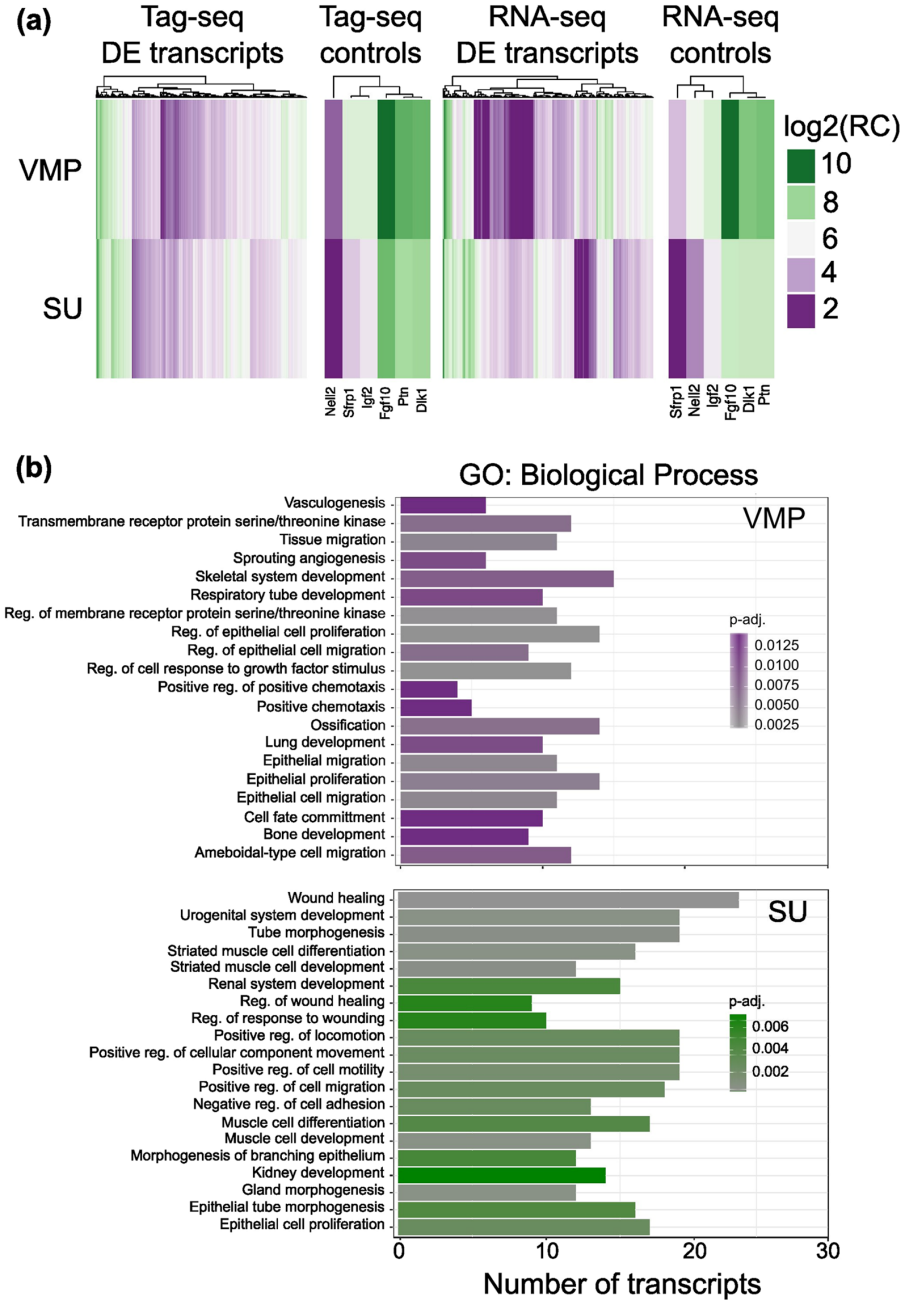
Differential expression of transcripts between VMP and SU, and Gene Ontology of pathways associated with tissue subsets. (**a**) Heatmap representing expression values of the 400 differentially expressed transcripts (M ≥ 1.5 and q > 0.9) between VMP and SU identified by both Tag-seq and RNA-seq, as well as previously published markers of VMP (Nell2, Sfrp1, Igf2, Fgf10, Ptn, Dlk1). The colour key represents log_2_-trimmed mean of M component normalized read counts (log2(RC)). Both Tag-seq and RNA-seq identified transcripts showing differential expression between SU and VMP (**b**) Gene ontology analysis of VMP and SU enriched transcripts. The figure shows the biological process group found with an FDR adjusted *P*-value < 0.05. Bar length represent the number of VMP and SU enriched transcripts in each group and the shade of colour the adjusted *P*-value for the enrichment. VMP showed enrichment of pathways related to regulation of epithelial proliferation, migration and response to growth factors, while the SU showed enrichment of pathways related to muscle development consistent with its tissue composition.

Visualisation of the 400 differentially expressed transcripts by heatmap supported the differential expression between VMP and SU, which was also evident in transcripts previously identified as VMP specific or enriched (Scube1, Nell2, Rspo2, Rspo3, Ptn, Igf2, Sfrp1, Fgf10)^8^ (Fig. 2a). Gene Ontology analysis identified regulation of epithelial cell proliferation, migration and growth factor response as associated with VMP enriched transcripts (Fig. 2b), as well processes involved in glycosaminoglycan binding and axon guidance. Molecular functions such as Wnt and Vegf protein binding as well as promoter DNA binding were also identified as significant in VMP enriched transcripts (Supplementary Figure 3). Gene Ontology analysis of SU identified several pathways associated with muscle development consistent with its tissue composition (Fig. 2b).

### Single cell RNAseq of mesenchymal subsets

**Figure 3:**
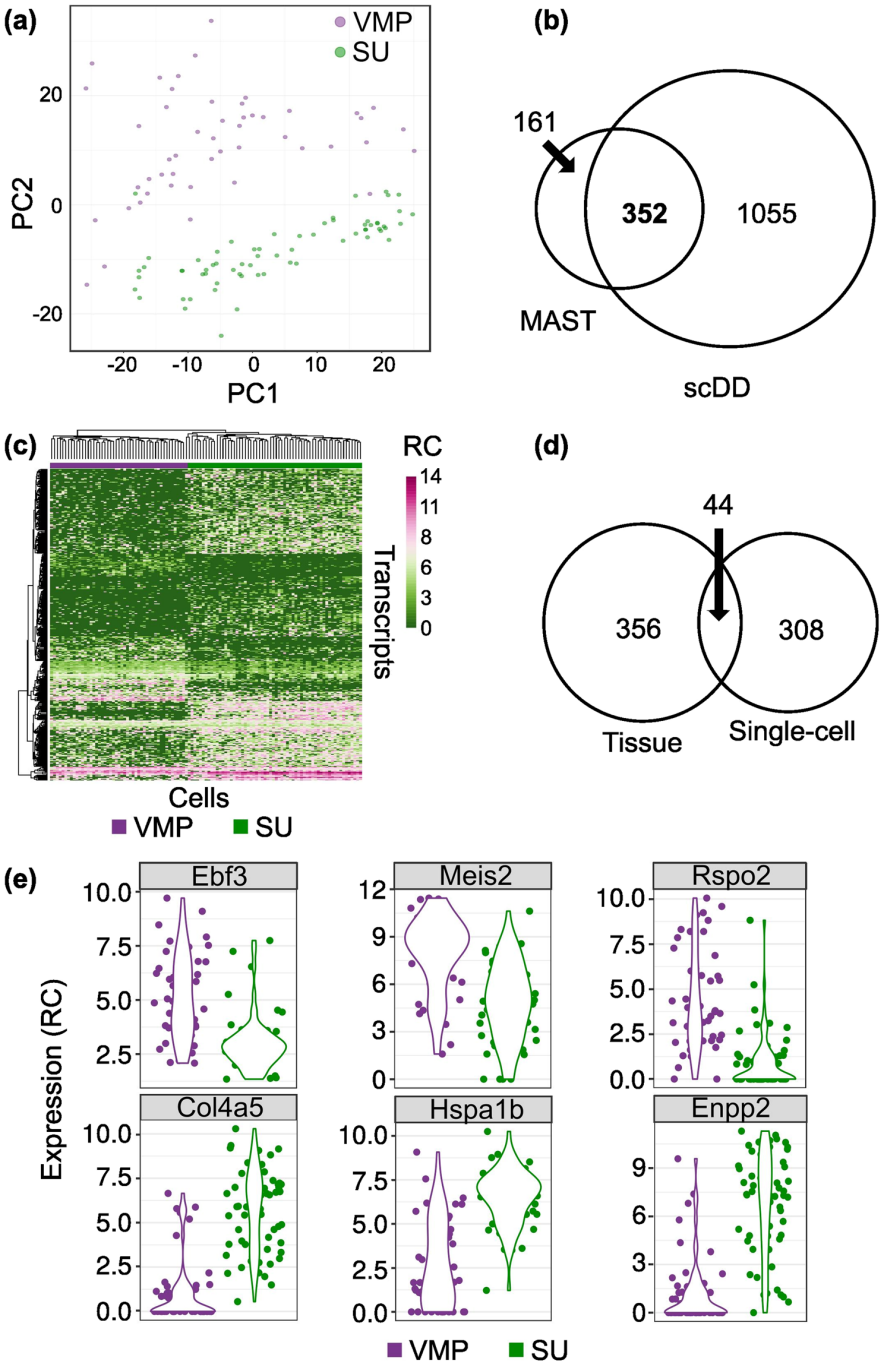
Single-cell RNA-seq of dissociated VMP and SU cells. Microdissected tissues were digested with collagenase to provide single cell suspensions for separation using a Fluidigm C1 microfluidic chip, and subsequent library preparation and sequencing. (**a**) PCA plot of whole transcriptome data distinguished VMP from SU cells. (**b**) Venn diagram showing the overlap between the DE transcripts identified by MAST (Benjamini-Hochberg adjusted *P*-value < 0.05) and the DE transcripts identified by scDD method (fold change > 1.5 and Benjamini-Hochberg adjusted *P*-value < 0.05). (**c**) Heatmap representing the log_2_ expression values (normalized read count + 1) (RC) of the common 352 DE transcripts between VMP and SU cells. (**d**) Venn diagram of DE transcripts identified by Tag-seq/RNA-seq and DE transcripts identified in scRNA-seq. 44 transcripts were common between both approaches. (**e**) Violin plots showing the distribution of log2 (normalized read count + 1) (RC) across VMP (top row) and SU (bottom row) cells for selected DE transcripts with adjusted *P*-value < 0.0001 identified by MAST and scDD.

To examine transcript expression and identify DE transcripts at single cell resolution, we performed scRNA-seq on dissociated cells derived from microdissected VMP and SU. VMP and SU single cells were isolated using a Fluidigm C1 chip, RNA-seq libraries were prepared and sequenced. scRNA-seq data was quality controlled to remove cells with low library size and low number of mapped genes as well as a high ratio of reads mapped to mitochondrial DNA and spike-in controls. The distribution of library size, number of mapped genes, proportion of reads mapped to mitochondrial DNA and proportion of reads mapped to spike-in controls are shown in Supplementary Figure 4a,b. Cell cycle was analysed in all cells and represented a median of 3.16% of gene expression variance (Supplementary Figure 5). We performed a PCA plot (Supplementary Figure 5c,d) and observed a clear separation of cells according to cell type (PC2 R^2^ = 0.58) but not according to cell-cycle stage (PC23 R^2^ = 0.17) indicating that cell cycle ha**s** a minor confounding effect. We have observed that VMP cells do not grow as primary cultures, but that SU stroma will grow *in vitro* (unpublished), and we suggest that differences between VMP and SU tissues may include factors related to proliferation, but that differences in cell cycle are a minor component of our data. In total 49 VMP and 62 SU single cells passed quality control and were used for further analysis. The landscape of cells in 2D space is shown by principal component analysis (PCA) and showed a separation between VMP and SU cell types demonstrating that dissociated cell populations retained their different tissue identities (Fig. 3a). Two algorithms (MAST^25^ and scDD^26^) were used to identify DE transcripts from scRNA-seq data. 513 and 1407 DE transcripts were identified by MAST and scDD respectively with 352 transcripts common to both (Fig. 3b). Visualization of the 352 DE transcripts by heatmap and hierarchical clustering showed a clear separation between VMP and SU cell populations (Fig. 3c). Figure 3d shows a Venn diagram of DE transcripts from tissue based analysis (400) compared to DE transcripts in scRNA-seq (352), which identified 44 transcripts as common to both. To further assess the effect of cell cycle status on DE transcript analysis, we compared the 352 DE transcripts to the list of cell cycle associated genes. A minority of DE transcripts (19, ~5%) were found to be cell cycle associated (Supplementary Figure 6a). We also identified DE transcripts between VMP and SU cells using MAST with or without adjusting for cell cycle. We found that correcting for cell cycle bias made a minor difference to the results (513 vs 578 transcripts; 453 common between analyses) (Supplementary Figure 6b). Comparison of the DE transcripts to the human foetal prostate transcriptome^19,24^ co-identified 27 transcripts suggesting that a high proportion (61%) of scRNA-seq identified DE transcripts are also expressed during human prostate development (Supplementary Figure 7). The distribution of expression of the transcripts was visualized by violin plot and demonstrated cell population specificity between VMP and SU cells (Fig. 3e and Supplementary Figure 8). Gene ontology analysis was performed on DE transcripts and significantly enriched terms were identified in the SU compartment only (Supplementary Figure 9). Pathways such as urogenital system development and functions such as Wnt pathway protein binding were identified supporting the gene ontology analysis performed on Tag- and RNA-seq whole tissue samples. We compared our data with an earlier SAGE analysis of VMP transcript expression, this identified a low percentage overlap (4%) but among the co-identified were Dlk1 and Ptn which were experimentally confirmed as VMP specific^20,27^ (Supplementary Figure 10).

### Analysis of cellular heterogeneity using single cell RNAseq

**Figure 4:**
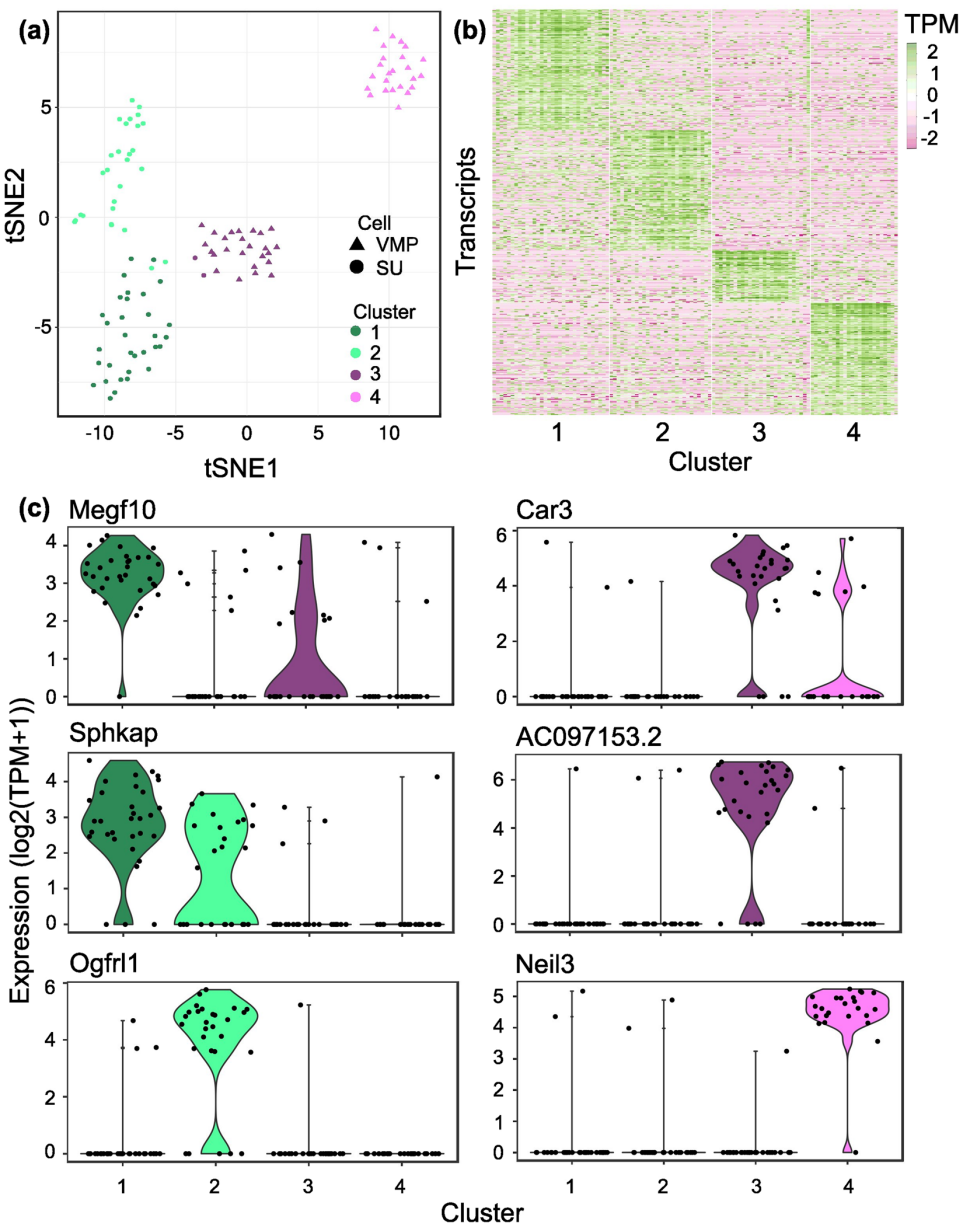
Identification and characterization of distinct cell subpopulations within VMP and SU tissue compartments. (**a**) tSNE analysis identified markers of cell subpopulations that separates both VMP and SU in to two distinct VMP and two distinct SU cell subpopulations labeled clusters 1 through 4 respectively. (**b**) Expression heatmap of most discriminatory cluster markers (avg_diff > 0 and AUC > 0.75) across VMP and SU cell populations. Expression values presented as log_2_ (TPM + 1) (TPM). (**c**) Violin plots showing expression of selected cluster marker genes across VMP and SU cell populations. Expression is presented as log_2_ (TPM + 1). Width of the violin plot indicates frequency of cells with that expression level.

We next performed a subset analysis using two algorithms (Seurat^28^ and SC3^29^) to determine whether VMP and SU cells were homogeneous or composed of subgroups. With both algorithms, single cells were organized into four distinct clusters (two VMP and two SU clusters, Fig. 4 & Supplementary Figure 11). tSNE analysis showed organization of cells into 4 distinct clusters in 2D space (Fig. 4a). This suggests that VMP and SU compartments are not homogeneous. Transcripts enriched within each of the four clusters were identified using the Seurat algorithm by first identifying the most variable genes between each cluster followed by a statistical ROC analysis to identify the transcripts differentially expressed between each of the four clusters. A total of 846 DE transcripts were identified between the four clusters with an AUC score > 0.75 and a power score > 0.4. Of these, 290 were classified as enriched for cluster 1 (SU cells), 294 were classified as enriched for cluster 2 (SU cells), 103 were enriched for cluster 3 (VMP cells) and 159 were enriched for cluster 4 (VMP cells). The expression of these transcripts were visualized by heatmap and showed a clear separation of the four cell clusters (Fig. 4b). Figure 4c and Supplementary Figure 12 shows the distribution of expression of representative transcripts from each of the four clusters by violin plot at single cell resolution.

### Validation of compartment specific transcript expression by qPCR

**Figure 5:**
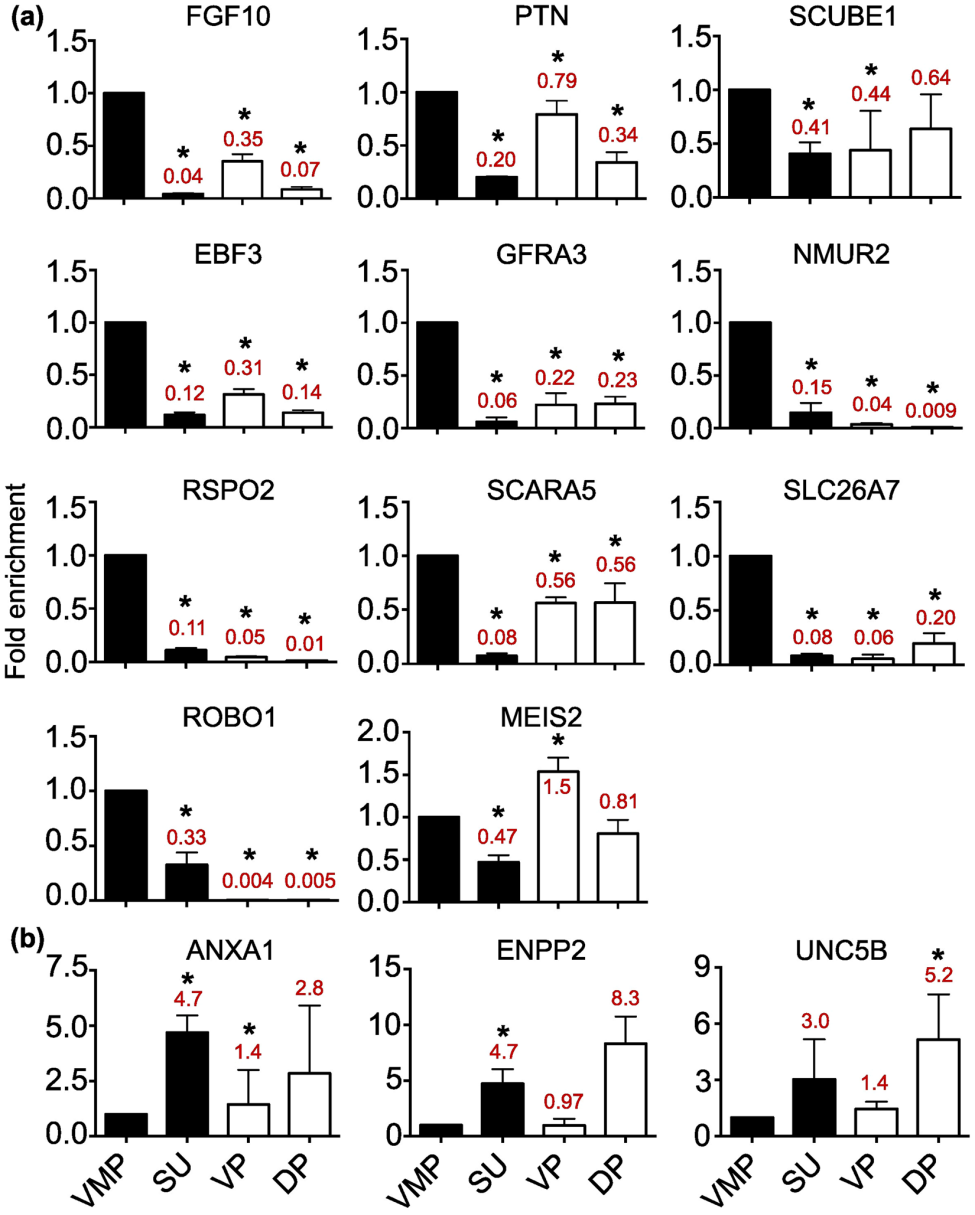
Validation of VMP- and SU-specific transcript expression in female and male P0 rat tissues. Quantitative real-time PCR (qPCR) showed significantly elevated levels of both control (Fgf10, Ptn and Scube1) and candidate (Ebf3, Gfra3, Nmur2, Rspo2, Scara5, Slc26a7, Robo1 and Meis2) VMP-specific transcripts versus SU. Fgf10, Ptn, Scube1, Ebf3, Gfra3, Scara5 and Meis2 were expressed in VP and DP, while Rspo2, Nmur2 and Slc26a7 showed low expression in VP and DP. SU candidate transcripts Anxa1, Enpp2 and Unc5b were enriched versus VMP. Data is represented as mean fold difference to VMP ± SD of duplicate biological replicates and duplicate technical replicates (n = 4). Significance was detected using One-way ANOVA with TUKEY multiple comparison *p < 0.05. Figures in red indicate fold difference compared to VMP.

To validate differential expression of VMP and SU enriched transcripts, we performed qPCR upon pooled microdissected tissues. In addition to VMP and SU tissues, we included ventral prostate (VP) and dorsal/dorsolateral prostate (DP) to compare expression between VMP and prostate lobes. Prostate tissue was composed of both mesenchyme and epithelia, thus mesenchyme-specific transcripts would be diluted due to the presence of epithelia in VP and DP. We examined 11 of our differentially expressed transcripts by qPCR in VMP, SU, VP and DP samples (Fig. 5), as well as a panel of known VMP enriched (Fgf10, Ptn, Scube1) or SU enriched (Aldh1a3, Wnt5a, Lef1 and Bmp4, Supplementary Figure 13) transcripts. Overall, our VMP differentially expressed transcripts were significantly enriched in VMP tissues versus SU tissues by qPCR. This validated our bioinformatic approaches for identification of VMP enriched transcripts. A subset of transcripts also showed VMP enrichment as well as expression in VP and DP. Our SU enriched transcripts outperformed known SU enriched markers upon validation by qPCR (Fig. 5 and Supplementary Figure 13).

### Immunohistochemical localisation of subset specific proteins Ebf3 and Meis2

**Figure 6:**
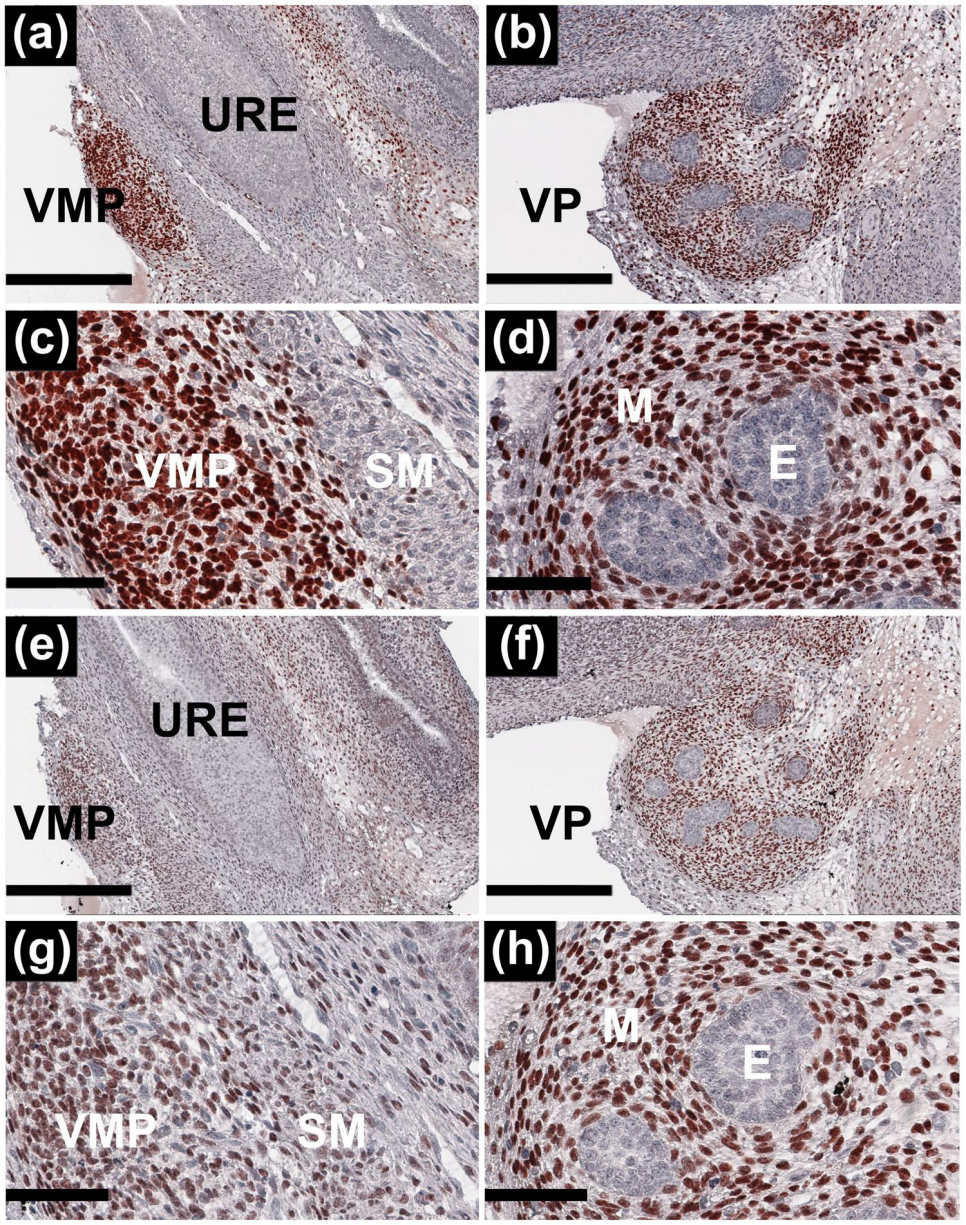
Immunohistochemistry of Ebf3 and Meis2 in female VMP and male prostate. To examine the distribution of Ebf3 and Meis2 in mesenchymal subsets in VMP and ventral prostate (VP) we performed immunohistochemistry using P0 rat female and male reproductive tracts. Panels (**a–d**) show Ebf3 expression; in female (**a and c**) and male (**b** and **d**). Ebf3 showed a highly selective distribution within VMP and VP mesenchyme, and was absent from smooth muscle (SM) and the urethra (**c**, **a and b**). Panels (**e–h**) show Meis2 distribution; in female (**e** and **g**) and male (**f** and **h**) mesenchyme. Urethral epithelia (URE) and prostatic epithelia (E) were negative for both Ebf3 and Meis2. Scale bars (**a**,**b**,**e** and **f**) = 600 µm. Scale bars (**c**,**d**,**g** and **h**) = 300 µm.

In order to determine whether the differentially expressed transcripts were cell subset specific, we examined protein expression by immunohistochemistry upon P0 female and male urethra focussing upon the prostate and VMP. We chose two VMP enriched markers, transcription factors Ebf3 and Meis2, and documented their expression in serial sections of female and male P0 rat urogenital sinus tissue (Fig. 6). We observed that Ebf3 was nuclear and largely homogeneously expressed in female VMP cells. Protein expression was markedly reduced in the SU versus the VMP cell compartments supporting our transcriptomic data. In male tissues, Ebf3 was nuclear and restricted to the mesenchymal cells of the developing ventral prostate with no expression in the epithelial cells of ventral prostatic buds. Meis2 showed a similar nuclear and mesenchymal cell specific expression pattern in both female and male tissues.

## Discussion

The ventral mesenchymal pad (VMP) is a subset of urogenital mesenchyme which has been shown to express potent morphogens and regulate prostate organogenesis^9,30^. Signalling from the VMP and urogenital sinus mesenchyme can re-specify epithelial fate^31^ and partially re-differentiate prostate tumour epithelium^32^. Recently, we identified Asporin (ASPN) as expressed within a subset of human prostate mesenchyme^20^, and showed that ASPN was a marker of prostate tumour stroma associated with disease progression^33^. Similarly, expression of VMP specific morphogens in cancer associated fibroblasts was able to reduce tumour growth in a human prostate tumour reconstitution model^34^. This demonstrates the significance of the VMP as a source of stromal-specific molecules with potent capacity to regulate epithelial growth and differentiation in both development and disease. We suggest that mesenchymal subsets are enriched for regulators and morphogens, as well as factors associated with paracrine signalling between mesenchyme and epithelium or juxtacrine signalling between mesenchymal subsets. Our studies are among the first to catalogue gene expression in inductive mesenchyme, and address cellular heterogeneity within the mesenchymal compartment.

In this study, our goal was to identify molecules specific to the VMP and to examine cell and tissue heterogeneity within the mesenchyme. We performed both Tag-seq and RNA-seq on VMP as well as an adjacent mesenchyme comprised of urethral epithelium and peri-urethral mesenchyme (termed SU). Comparison of VMP Tag/RNA-seq libraries to SU libraries identified 400 transcripts that were differentially expressed between the two compartments. Among these were several transcripts identified as VMP-specific in a previous SAGE study^8^, which served as controls for our analysis (Fig. 2). The identification of particular transcripts with different techniques supports the reproducibility of our results. Gene set enrichment analysis of VMP enriched transcripts were associated with biological signalling pathways related to epithelial cell migration, differentiation and proliferation consistent with the function of the VMP as a potent regulator of epithelial cell development. The VMP is part of a condensed area of mesenchyme that encircles the urethra and which overlies peri-urethral mesenchyme. It appears that there was significant expression of regulatory pathways in both the VMP and peri-urethral mesenchyme. A recent ontology analysis has described the distribution of mesenchymal subregions^35^ and our results provide molecular characterisation of these subsets. While there is paracrine signalling between mesenchyme and epithelium, we speculate that there is also juxtacrine signalling between different mesenchymal compartments. It may be possible to bioinformatically identify ligands and receptors with reciprocal expression between mesenchymal subsets. Differences between VMP and SU were confirmed using scRNA-seq analysis, which showed distinct gene expression between these compartments and co-identified 44 transcripts observed in the tissue-based analysis. Deeper analysis of the scRNA-seq data determined that both VMP and SU were comprised of 2 subsets (Fig. 4). This analysis also identified subset specific markers, and suggested that there was low heterogeneity within the VMP and SU compartments. At present, we do not know the functional significance of the two subgroups that make up the VMP and SU compartments, however, this heterogeneity will be important to consider when using tissue-specific promoters for gene targeting as many promoters will be active in a proportion of cells rather than throughout all cells in the tissue.

Several of the markers identified in VMP and SU were validated by qPCR and simultaneously examined for their expression in male developing prostate. We propose that VMP mesenchyme provides a simpler model for the identification of mesenchyme specific molecules since it lacks branching epithelia. Inclusion of these in whole tissue transcriptomics yields more complex data in which it is difficult to deconvolve mesenchyme specific molecules. Comparison between male and female mesenchyme may be used to identify sexually dimorphic gene expression and regulation by androgens and the androgen receptor, and we chose to focus upon mesenchyme-specific expression rather than sexually dimorphic expression. We note that some of our markers exhibit differences between male and female mesenchyme which could be validated in future studies. This is important, since androgen action within the mesenchymal compartment regulates both prostate and genital tubercle growth, and we propose that identification of mesenchyme specific molecules is a first step in the discovery of such factors.

We identified Meis homeobox 2 (Meis2) and early B-cell factor 3 (Ebf3) as specific to the VMP compared to SU, and also expressed in prostatic mesenchyme. Meis2 belongs to the TALE homeobox protein family and is a regulator of transcription^36^. Meis2 has been identified as essential for the development of cardiac, orofacial, gastro-esophageal and neural tissues^37–39^. Ebf3 is a DNA-binding transcription factor which is involved in the development of bone and neural tissues^40,41^. Here we have established specific expression and nuclear localisation of Meis2 and Ebf3 in developing prostate mesenchyme and are the first to associate these transcription factors with prostate development and expression within mesenchymal subsets. We propose that these molecules can be used as specific markers of mesenchyme or stroma and could be used to estimate the abundance of stroma vs epithelium in tissues of mixed cellular composition.

In conclusion, we present a high-resolution transcriptomic analysis of inductive prostate mesenchyme that has documented limited cellular heterogeneity within subsets and identified markers and pathways expressed in mesenchyme during early prostate organogenesis.

## Methods

### Animal and tissue collection

Wistar rats were housed under a 12-hour light/dark cycle and maintained on standard laboratory diet, the study was performed under MUHC animal protocol number 2015–7670, approved by the McGill University Facility Animal Care Committee (FACC). Newborn (P0) pups were sacrificed by cervical dislocation and decapitation (in accordance with local guidelines and regulations), followed by removal of the urogenital tract and microdissection of the urethra into VMP and SU components using a Leica MZ6 dissection microscope.

### Tag-sequencing library preparation

Pools of microdissected tissues (VMP and SU) from over 100 animals were processed for digital gene expression Tag-profiling using NlaIII and a protocol provided by Illumina followed by sequencing on an llumina GAIIX (1 × 50 SE) at one lane per sample (25–30 m reads). PolyA + RNA was purified, cDNA synthesised, digested with NlaIII and ligated to Adaptor 1 (containing an Mme1 site). Samples were digested with Mme1, ligated to Adaptor 2, and PCR amplified, followed by gel electrophoresis and purification of 85 bp fragments that were sequenced. DNA sequencing was carried out in the GenePool genomics facility in the University of Edinburgh.

### Single-cell RNA-sequencing library preparation

Dissociated cells derived from collagenase digestion of pools of microdissected VMP and SU using collagenase 1 A at 2 mg/mL concentration (Sigma-Aldrich, Missouri, USA) for 60 minutes at 37 °C. Dispersed fibroblasts were separated from epithelia and tissue clumps by centrifugation through a 0.7μm cell strainer (Falcon® Corning, Corning, New York, USA).

Cell suspensions were centrifuged for 10 minutes at 500 g and resuspended in LIVE/DEAD Cell Viability/Cytotoxicity Assay for mammalian cells (ThermoFisher, L-3224). After a 10-minute incubation at room temperature, cells were centrifuged and resuspended in Cell Wash Buffer (Fluidigm). Cell concentration, size and viability were verified on hemocytometers (Incyto DHC-N01–5) through bright field, GFP and RFP on a EVOS FL Auto microscope (ThermoFisher). Single cell RNA libraries were constructed according to the Fluidigm protocol using C1 to generate libraries for RNA sequencing (PN 100–7168). Briefly, full length mRNA-seq libraries were generated from single-cells captured on the Fluidigm C1 platform using SMARTer Ultra Low RNA Kit (P/N 634936 Clontech). ERCC RNA Spike-In mix (P/N 4456740 ThermoFisher) was added to the lysis mix for normalization and quality control purposes. Full length cDNAs were converted into sequence ready libraries using Nextera XT DNA Sample Preparation Kit (P/N FC-131–1096 Illumina), and sequenced on an Illumina HiSeq2000/2500 with paired-end 100/125 option. In parallel, for every sample, sequencing libraries from bulk cells (200 cells) using 5 ng of purified total RNA, and a negative control were run on a thermocycler (T100 BioRad).

The Fluidigm C1 platform captured 52 and 70 single cells from VMP and SU respectively. The average full length cDNA yield/min/max were 6.06ng(+/−0.12)/2.25ng/14.81ng for VMP and 8.51ng(+/−0.21)/2.55ng/26.6ng for SU. Libraries from 52 VMP and 63 SU single cells were sequenced.

Cells from the cell suspensions were processed to provide a ‘bulk’ comparator for single cell studies.

### Tag-sequencing read alignment

Raw sequencing reads were trimmed to 17 bp to remove adaptor sequences and restriction digestion sites. Reads were quality controlled using FastQC^42^ to keep only reads with a mean quality score of 20 and above. Reads were aligned to the rat genome (Ensembl Rnor_6.0) using the Bowtie2 algorithm (default settings)^43^. Reads aligned to random contigs and mitochondrial DNA were removed and only uniquely mapped reads with a mapping quality >=25 were used for further analysis.

### Single-cell RNA-sequencing read alignment

Raw paired-end reads were trimmed using Trimmomatic v0.33^44^, to a minimum length of 30 nucleotides. Illumina Nextera XT adapters were removed in palindrome mode. A minimum Phred quality score of 30 was required for the 3′ end. Single end reads as well as paired end reads failing previous minimum quality controls were discarded. Individual read groups were aligned, using TopHat^45^ first against the rat transcriptome as defined by the Ensembl gene models *version 83*, with default parameters and the remaining unmapped genes to the Ensembl *Rnor_6*.*0* reference rat genome from Illumina iGenomes web site. Trimming rates and insert length were controlled on each read group based on metrics reported by Trimmomatic, and Picard v1.128 respectively.

Aligned reads from multiple read groups belonging to the same sample were indexed, sorted and merged using sambamba v0.5.1^46^, a faster implementation of the Samtools algorithms. Amplification duplicates were removed using Picard v1.128.

Various quality controls from the RNA-SeQC package were used^47^, including the genes detected, mapping rates, duplication rates, and intronic rate, based on metrics collected for each sample used.

### Read count quantification, normalization and differential gene expression

Read counts were quantified using the summarizeOverlaps function from the GenomicAlignments R package^48^. Transcripts with a read count of 0 in both samples were removed. EdgeR^49^ was used to perform TMM normalization and only transcripts with counts per million (cpm) > 1 were used for differential analysis of genes. The NOISeq package^23^ was used to screen differentially expressed genes between VMP and SU tissues. Genes with a *q*-value of >= 0.9 were considered differentially expressed.

### Gene Ontology enrichment analysis

Gene Ontology (GO) enrichment analysis was conducted using the clusterProfiler R package^50^ on the VMP and SU enriched genes. Ontology terms with an FDR < 0.05 were considered significant.

### Single-cell RNA-sequencing normalization, differential gene expression and subpopulation analysis

The Scater package^51^ was used for quality control and normalization. Low quality cells were filtered out based on library size, number of genes detected, proportion of reads mapped to mitochondrial genome and the ratio of reads mapped to spike-ins. Cells were removed if they met any of the following criteria: a median absolute deviation (MAD) value of less than 3 for library size, a MAD value of less than 3 for number of mapped genes, a MAD value of greater than 3 for the ratio of reads mapped to mitochondrial DNA and a MAD value of greater than 3 for the ratio of reads mapped to spike-in control DNA. The numbers of cells meeting these criteria are detailed in Supplementary Figure 4b. Genes expressed by less than 20 cells were discarded. Gene expression was normalized using spike-ins. Differentially expressed genes were identified using the MAST^25^ and scDD^26^ R packages. Prior to subpopulation identification normalized read counts were converted to TPM and analysis was performed using the Seurat and SC3 R packages^28,29^. For both packages, marker genes were identified using a ROC test. All markers with an AUC < 0.75 and power < 0.4 were removed.

### RNA extraction and quantitative real-time PCR

Total RNA was extracted from pooled tissues using Qiazol followed by the RNeasy^TM^ Mini kit (Qiagen, Venlo, Netherlands) following manufacturer’s instructions. Complementary DNA synthesis was performed using the High Capacity cDNA Reverse Transcription kit (Applied Biosystems- ThermoFisher Scientific, Massachusetts, USA) and qPCR was performed on an ABI 7500 Fast machine using SYBR Select Mastermix (ThermoFisher Scientific, Massachusetts, USA). Transcript abundance was normalized to four housekeeping genes; Gapdh, Tbp, Gusb and Mt-atp6. Primers used are provided in Table 1.

**Table 1:** Summary of primers used for qPCR.

Gene	Forward primer (5′–3′)	Reverse primer (3′–5′)
Fgf10	GGGAAACTCTATGGCTCAAAAG	TGCCACATACATTTGCCTGC
Ptn	GCCTCAAGCGGAATCAAAGA	ATCCTGCTTGCTGATGTCCT
Scube1	CCTATGACGAGGACTACCAG	TCTCCTGATGGTTCTCCGA
Ebf3	GCAACACTCCAGCACACACT	ATTGCGACTGTAGCCGACTT
Gfra3	CACCCTATGGACATCCTCG	CATGGCAGTCCCAATTAGC
Nmur2	CCTTGAGGCGAACAAAGTG	AGGACCAAGACAAACAGCA
Rspo2	GGAGAGTGTCTGCATTCCT	TATTCTGCATCTTGCACATCTG
Scara5	CAACGGCTCCATCTTTGAG	TTGTGACATGGACCATCCT
Slc26a7	TTGGTCCTGAATTGCAGTG	CTTGCAATCAAGGTAGAGCTC
Robo1	AGGTTTGGTGTCTCGGGAAT	AGCGGATTCCAGGAACAAGA
Meis2	TGACCTCGTGATTGATGAGAG	AGGATGAAGGGTTGTGGTC
Anxa1	AAAGGTGTGGATGAGGCAAC	TATGCTGCCTTGATCTGCTG
Enpp2	CATTCCAGGTAATATCCTTGTTCAC	CCCTCTTAATTCGACTTGCTG
Unc5b	AGGTACCCTTGGATCATGAG	TCTTGAGCCATTCCACCTC
Aldh1a3	CTACAACGCATTCTATGCAC	AAAGCGTATTCACCTAGTTCTC
Wnt5a	CAAGGGCTCCTATGAGAGC	GCCAGGTTGTATACTGTCCT
Lef1	TAGCAGACATCAAGTCATCCT	GTGTTTGTCTGACCACCTC
Bmp4	ATCACGAAGAACATCTGGAG	CTCATTCTCTGGGATGCTG
Gapdh	ATGACTCTACCCACGGCAAG	GATCTCGCTCCTGGAAGATG
Tbp	GCTGAATATAATCCCAAGCGGT	TGTGCACACCATTTTCCCAG
Gusb	TGGTATAAGATGTACCAGAAGCC	AGGTGGATCCTCATGAAGC
Mt-atp6	TGAGCCCTAATAATTGTATCCC	GAAGCCCTAGAAGGTTGGT

### Immunohistochemistry

Immunostaining of Ebf3 and Meis2 on serial sections of female and male rat P0 urogenital sinus tissue (isolated as per^20^) was performed as per^24^ using Ebf3 IgG (Clone 8D6, mouse monoclonal, Novus Biologicals, Littleton, Colorado, USA; dilution 1:1000) and Meis2 IgG (Clone 63-T, mouse monoclonal, Santa Cruz Biotechnology, Santa Cruz, USA; dilution 1:750). Primary antibody was omitted to serve as a negative control. Images were taken with an Aperio Slide Scanner (Leica, Wetzlar, Germany).

### Data Availability

All data generated by this work are available in GSE103011. Differentially expressed transcripts, Gene Ontology and transcript comparisons are provided in a supplementary data file.

## Electronic supplementary material

Supplementary Figures

Supplementary data
