# Supplementary material for: Identification of genes expressed in a mesenchymal subset regulating prostate organogenesis using tissue and single cell transcriptomics
Source: Sci Rep. 2017 Nov 27;7:16385. doi: 10.1038/s41598-017-16685-8 (PMC5703996; doi:10.1038/s41598-017-16685-8)
Supplement: Supplementary file 1 — Supplementary Figures [file 41598_2017_16685_MOESM1_ESM.pdf]

Supplementary Data for;

Identification of genes expressed in a mesenchymal subset regulating prostate organogenesis using tissue and single cell transcriptomics

Nadia Boufaied, Claire Nash, Annie Rochette, Anthony Smith, Brigid Orr, O. Cathal Grace, Yu Chang Wang, Dunarel Badescu, Jiannis Ragoussis[3], and Axel A. Thomson.

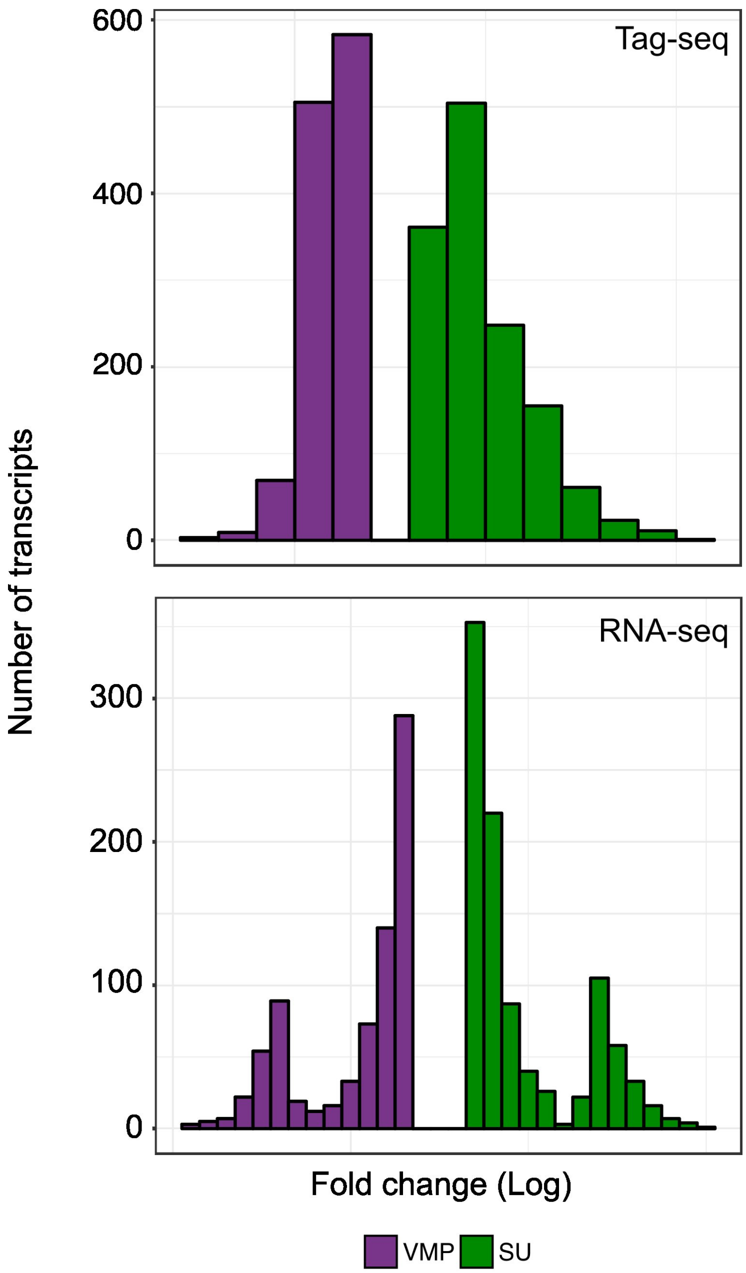

Supplementary Figure 1 Distribution of fold differences of differentially expressed transcripts between VMP and SU tissues with Tag- and RNA-sequencing. Histograms show the number of VMP differentially expressed (DE) transcripts (purple) or SU DE transcripts (green) against $\log_2$ fold change (VMP over SU or SU over VMP respectively) for DE transcripts identified by Tag-seq and RNA-seq.

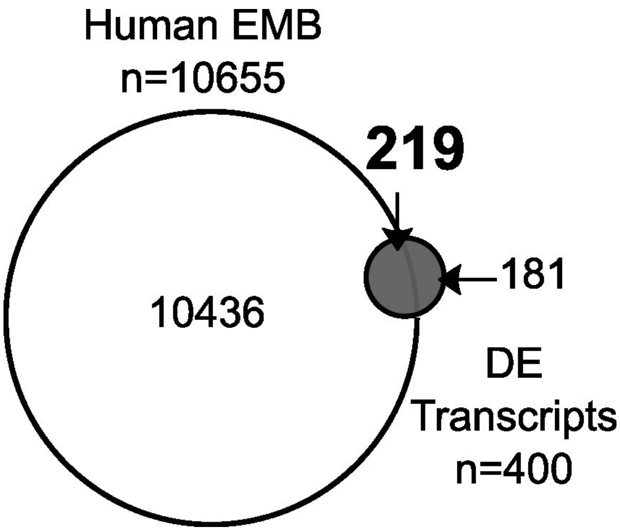

Supplementary Figure 2 Identification of differentially expressed transcripts between VMP and SU tissues, and human foetal prostate development. Venn diagram illustrating the overlap of differentially expressed transcripts (DE transcripts) with the human foetal prostate transcriptome (human EMB) [1, 2].

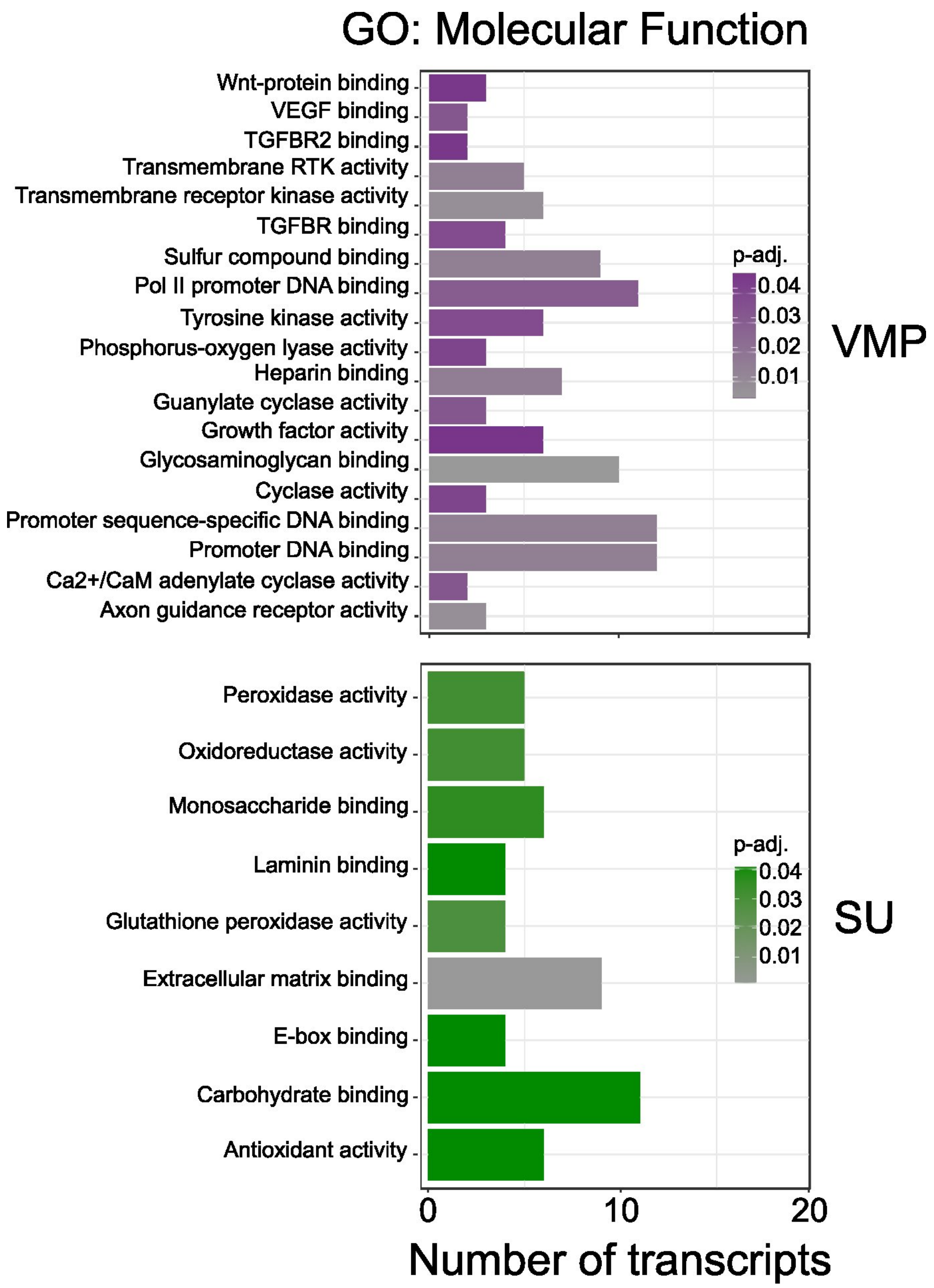

Supplementary Figure 3 Gene ontology analysis of VMP and SU enriched transcripts commonly identified by Tag- and RNA-sequencing. Histograms show the molecular function GO terms with an FDR adjusted *P*-value < 0.05. Shading of bars represents exact p-adj. value.

(a)

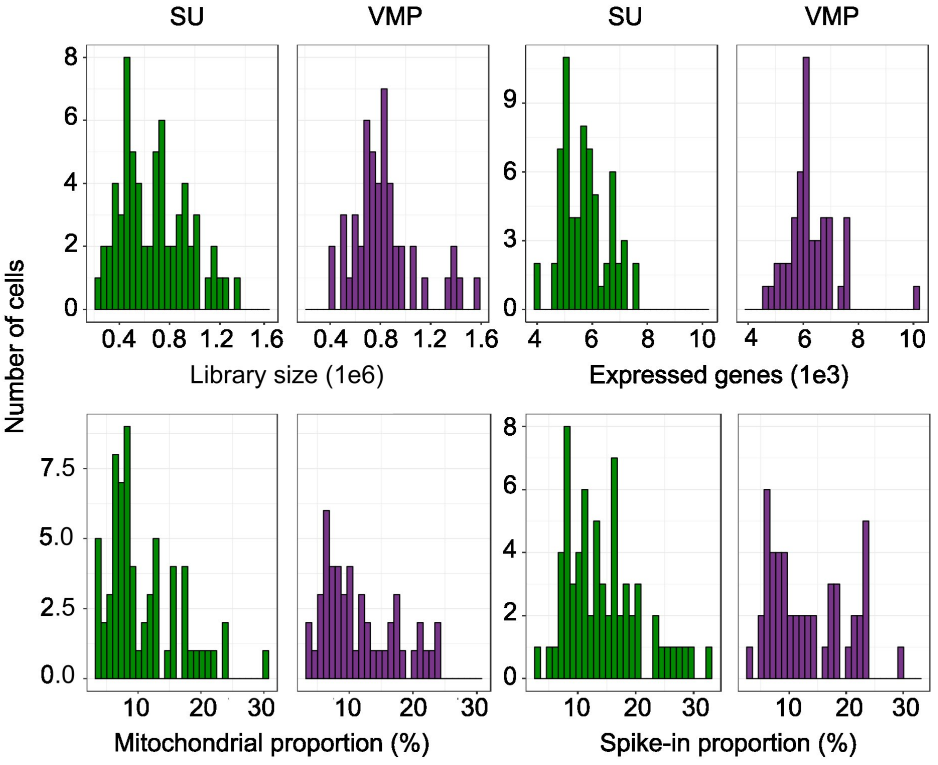

(b)

Feature	VMP	SU
Unassigned reads	295995	347657
Assigned reads	2651257	2847011
Unique reads	704364	779627
Spike-in reads	96015	96235
Mitochondrial reads	52959	70522
Genes	5544	6223
Cells used	**49**	**62**

Supplementary Figure 4 Quality control of VMP and SU single cell RNA-sequencing data. **(a)** Histograms show the number of cells against library size (per million), number of reads mapped to genes (per thousand), percentage of reads mapping to mitochondrial DNA and percentage of reads mapping to spike-in control DNA. SU cells are presented in green and VMP cells are presented in purple. **(b)** Summary table of numbers of reads, genes and total number of cells used following filtering for VMP and SU cells.

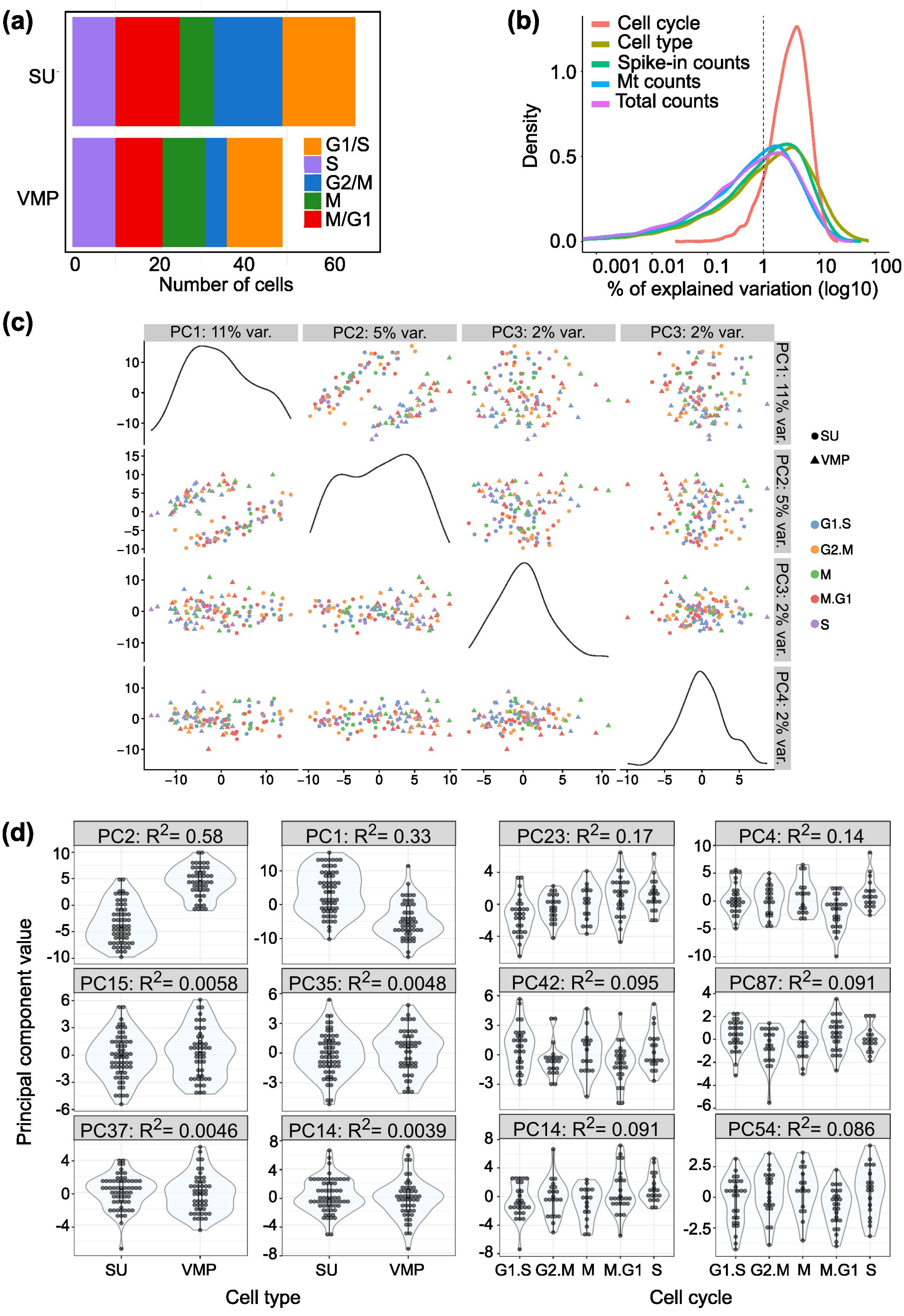

(a)
SU
VMP
G1/S
S
G2/M
M
M/G1
0
20
40
60
Number of cells
(b)
Cell cycle
Cell type
Spike-in counts
Mt counts
Total counts
Density
1.0
0.5
0.0
0.001
0.01
0.1
1
10
100
% of explained variation (log10)
(c)
PC1: 11% var.
PC2: 5% var.
PC3: 2% var.
PC3: 2% var.
PC4: 2% var.
SU
VMP
G1.S
G2.M
M
M.G1
S
(d)
PC2: R^2= 0.58
PC1: R^2= 0.33
PC23: R^2= 0.17
PC4: R^2= 0.14
PC15: R^2= 0.0058
PC35: R^2= 0.0048
PC42: R^2= 0.095
PC87: R^2= 0.091
PC37: R^2= 0.0046
PC14: R^2= 0.0039
PC14: R^2= 0.091
PC54: R^2= 0.086
Principal component value
SU
VMP
G1.S
G2.M
M
M.G1
S
Cell type
Cell cycle

Supplementary Figure 5 Confounding factors analysis of VMP and SU single cell RNA-sequencing data. **(a)** Histogram detailing the number of cells in each cell cycle phase. A phase-specific score was calculated and cells were assigned to a phase as per [3]. **(b)** Density plot of the percentage of explained variation for each factor (cell-cycle, cell type, spike-in counts, mitochondrial gene counts (Mt counts) and total read counts (Total counts) across all genes. Marginal R^2 for each variable was computed when fitting a linear model regressing read count for each gene against that variable. Each curve represents the distribution of percentage of variance across all genes for each factor. The median % of variance for cell-cycle, cell type, spike-in counts, Mt counts and Total counts was 3.16, 0.979, 0.754 and 0.681 respectively. **(c)** PCA visualization of VMP (triangle) and SU (circle) cells where each cell is coloured according to its cell cycle stage. **(d)** Principle components (PC) correlated with cell cycle. PC are ranked according to their R^2 from linear model regressing PC values against cell cycle. Var. = variance.

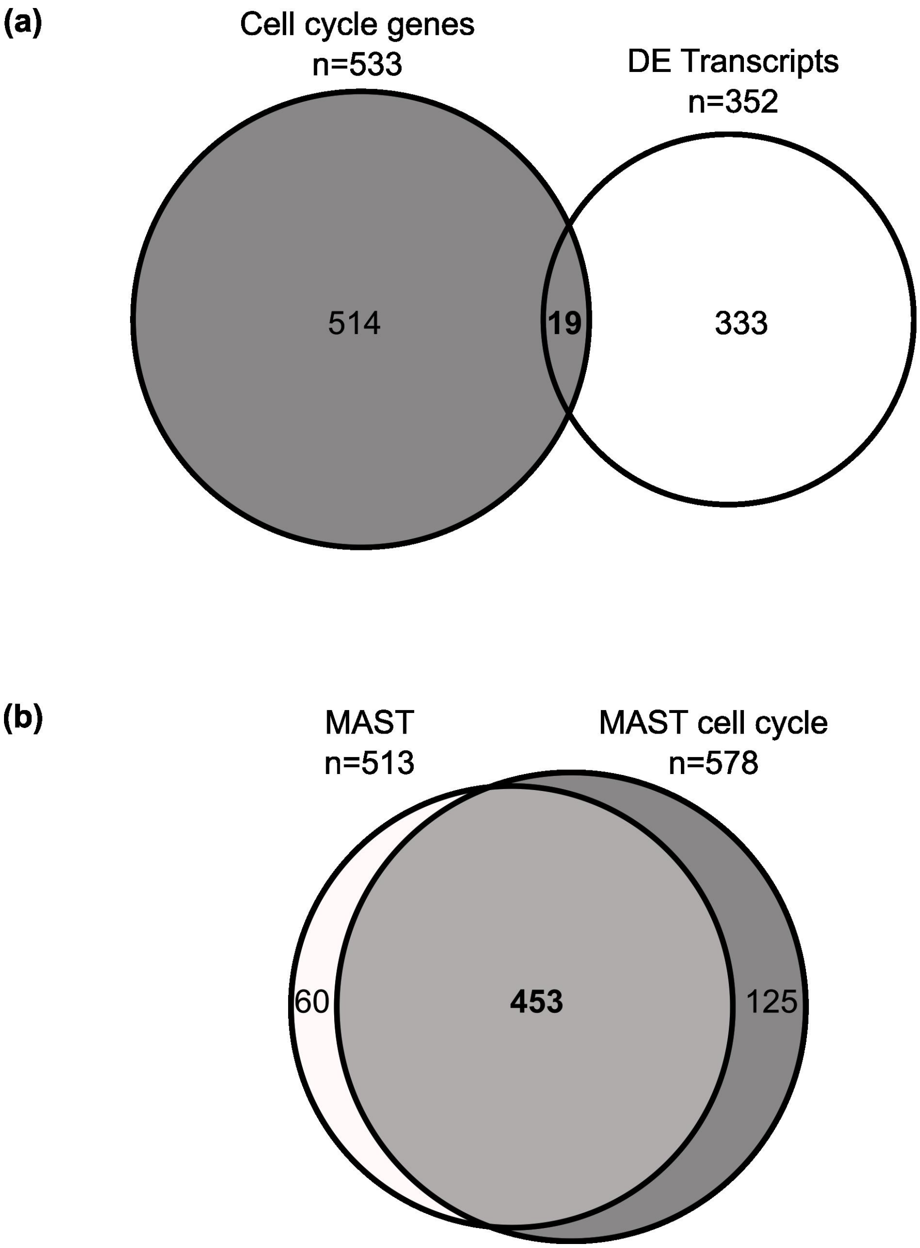

Supplementary figure 6 Cell cycle bias analysis. **(a)** Identification of differentially expressed transcripts associated with cell cycle. Venn Diagram illustrating the overlap of differentially expressed transcripts (DE Transcripts) with cell cycle- associated genes (Cell cycle genes). **(b)** Identification of differentially expressed transcripts between VMP and SU cells using MAST with and without cell cycle adjustment. Venn diagram showing the differentially expressed transcripts identified by MAST (MAST), and MAST corrected for cell cycle bias (MAST cell cycle). There was a high degree of overlap between the two analyses, and a low percentage of cell cycle-specific transcript expression.

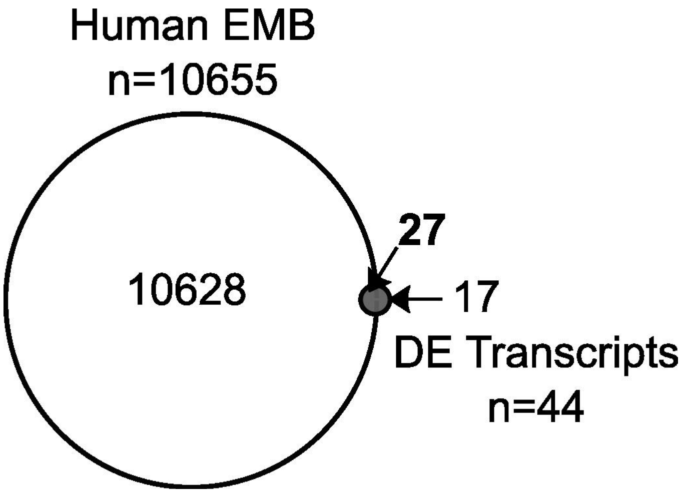

Supplementary Figure 7 Identification of differentially expressed transcripts between VMP and SU single cells with human foetal prostate tissue. Venn diagram illustrating the overlap of differentially expressed transcripts (DE Transcripts) with the human foetal prostate transcriptome (Human EMB) [1, 2].

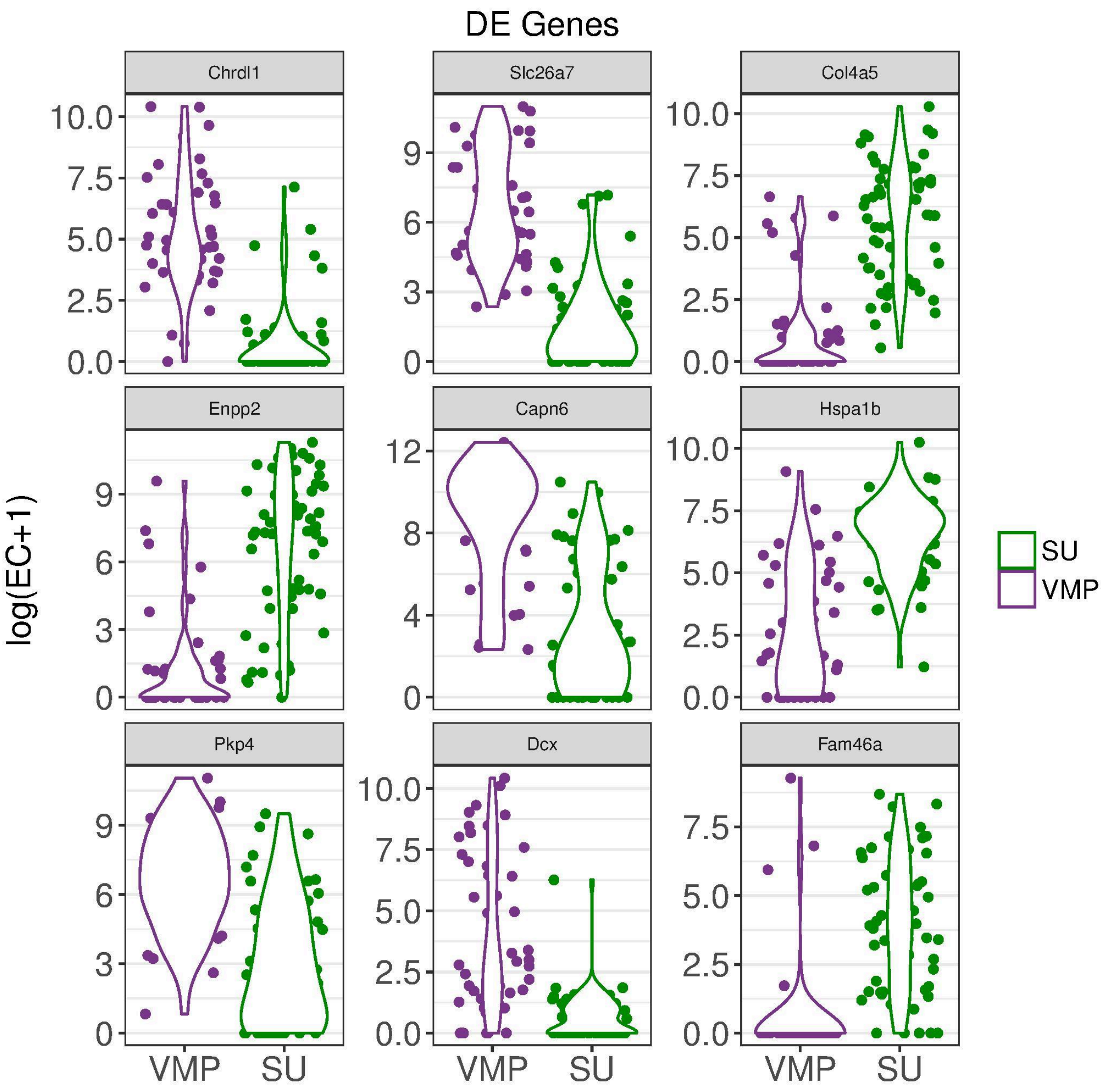
DE Genes
Chrdl1
Slc26a7
Col4a5
Enpp2
Capn6
Hspa1b
Pkp4
Dcx
Fam46a
log(EC+1)
VMP
SU
SU
VMP

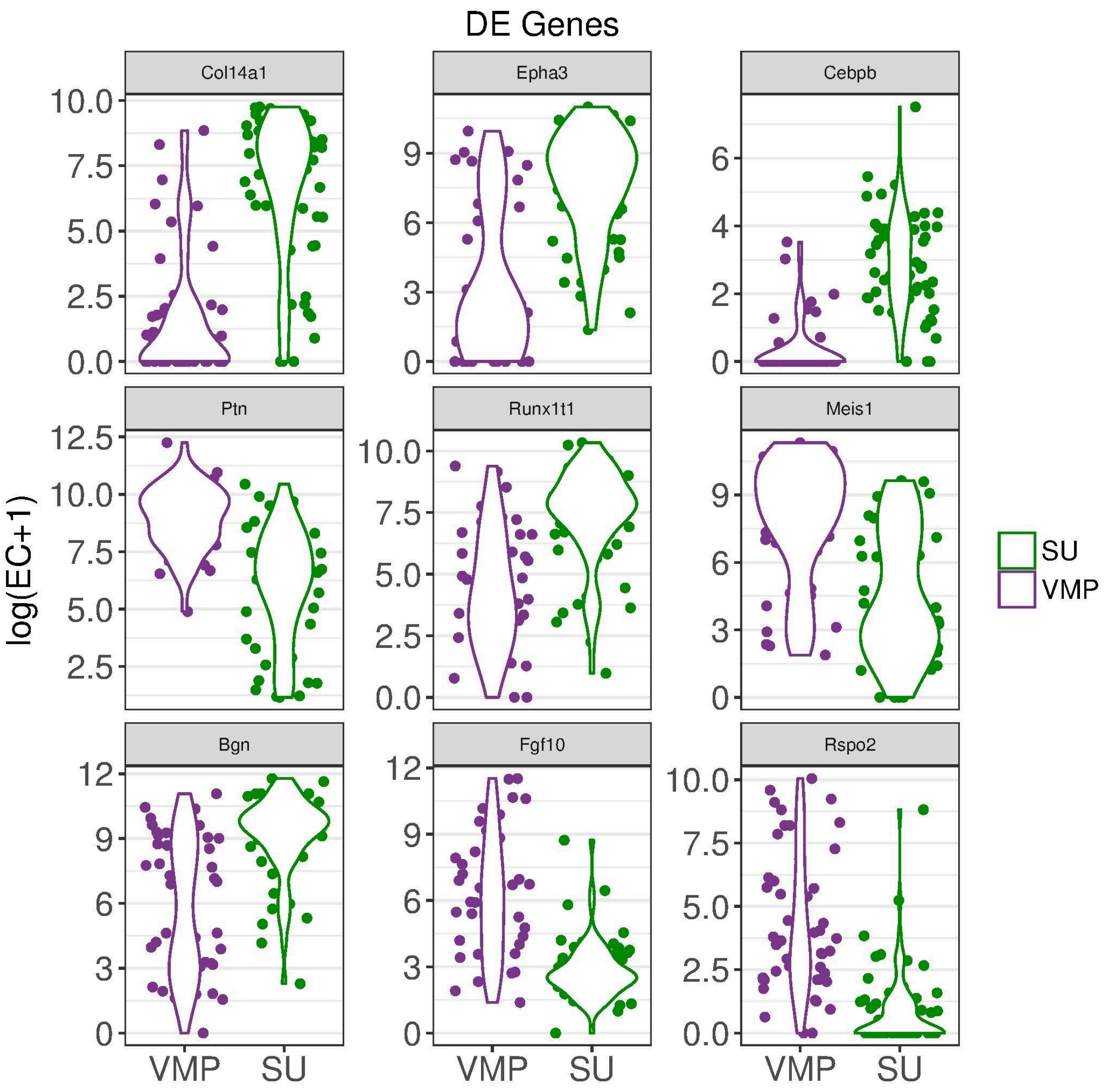
DE Genes
Col14a1
Epha3
Cebpb
Ptn
Runx1t1
Meis1
Bgn
Fgf10
Rspo2
log(EC+1)
VMP
SU
SU
VMP

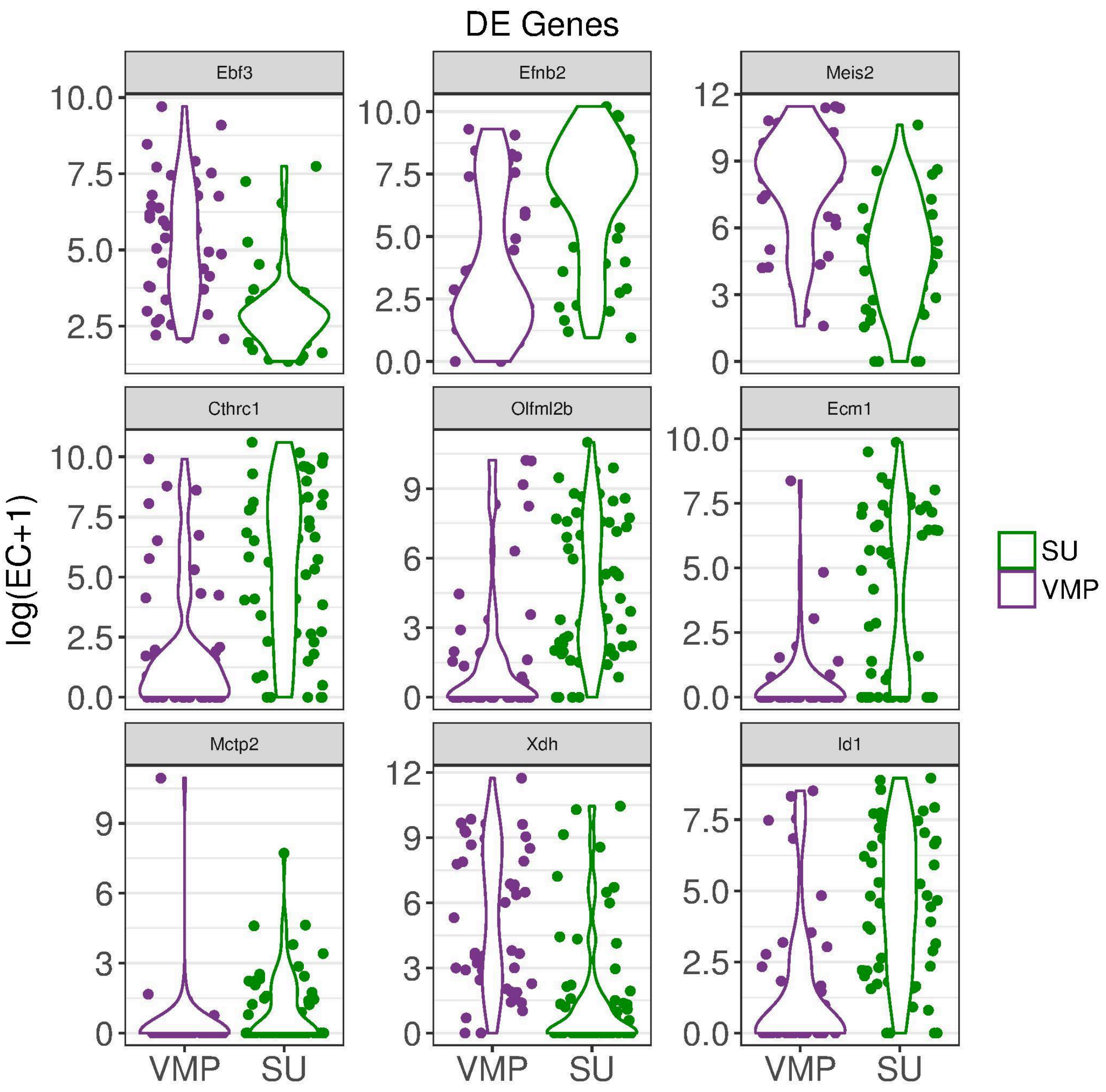
DE Genes
Ebf3
Efnb2
Meis2
Cthrc1
Olfml2b
Ecm1
Mctp2
Xdh
Id1
log(EC+1)
VMP
SU
SU
VMP

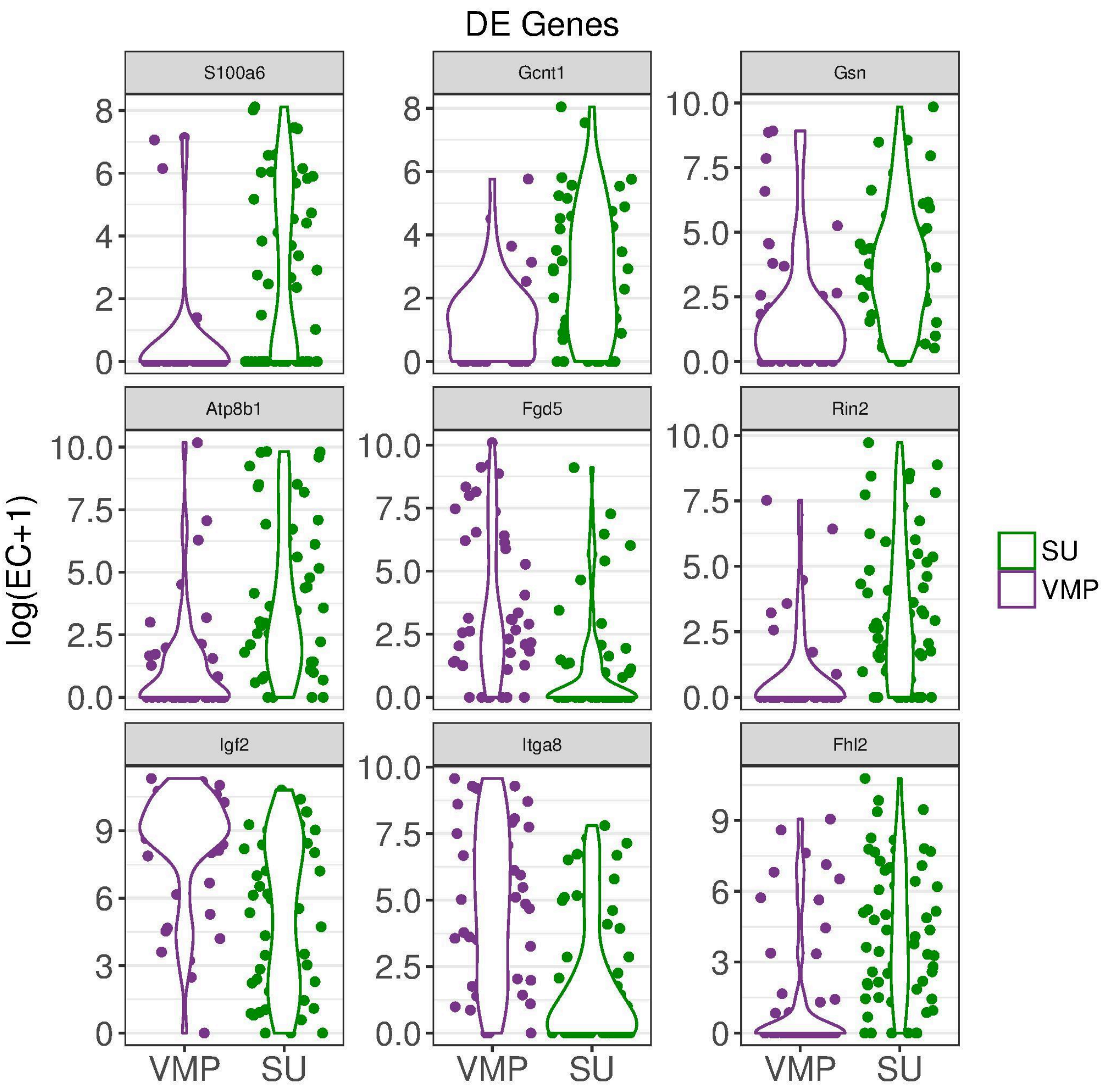
DE Genes
S100a6
Gcnt1
Gsn
Atp8b1
Fgd5
Rin2
Igf2
Itga8
Fhl2
log(EC+1)
VMP
SU
SU
VMP

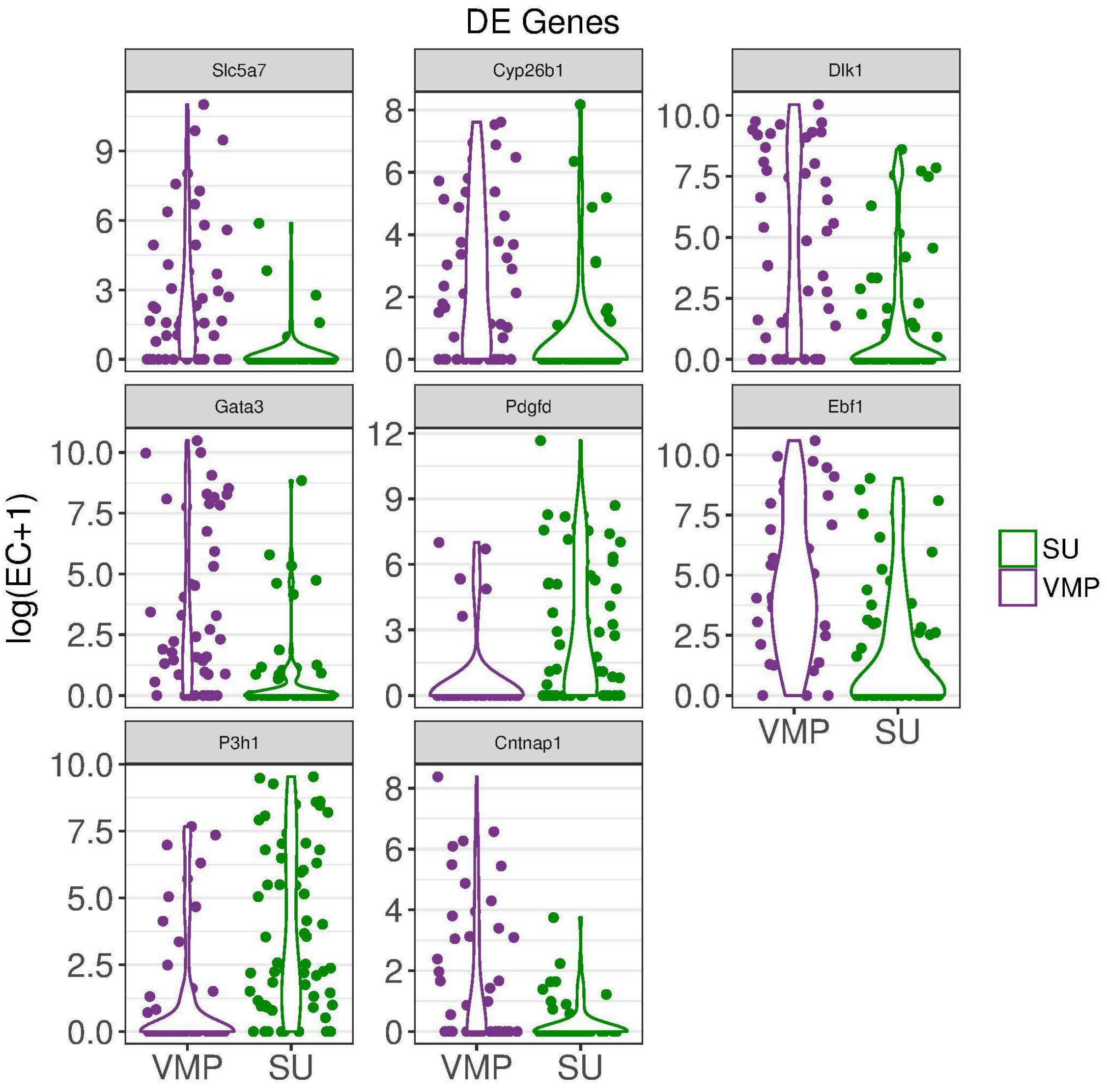

Supplementary Figure 8 Frequency distributions of expression of differentially expressed transcripts in scRNA-seq data commonly identified in Tag-seq, RNA-seq and scRNA-seq representing markers of VMP and SU cell populations. Expression is presented as log_2 read counts + 1 (log(EC+1)). Width of the violin plot indicates frequency of cells with that expression level.

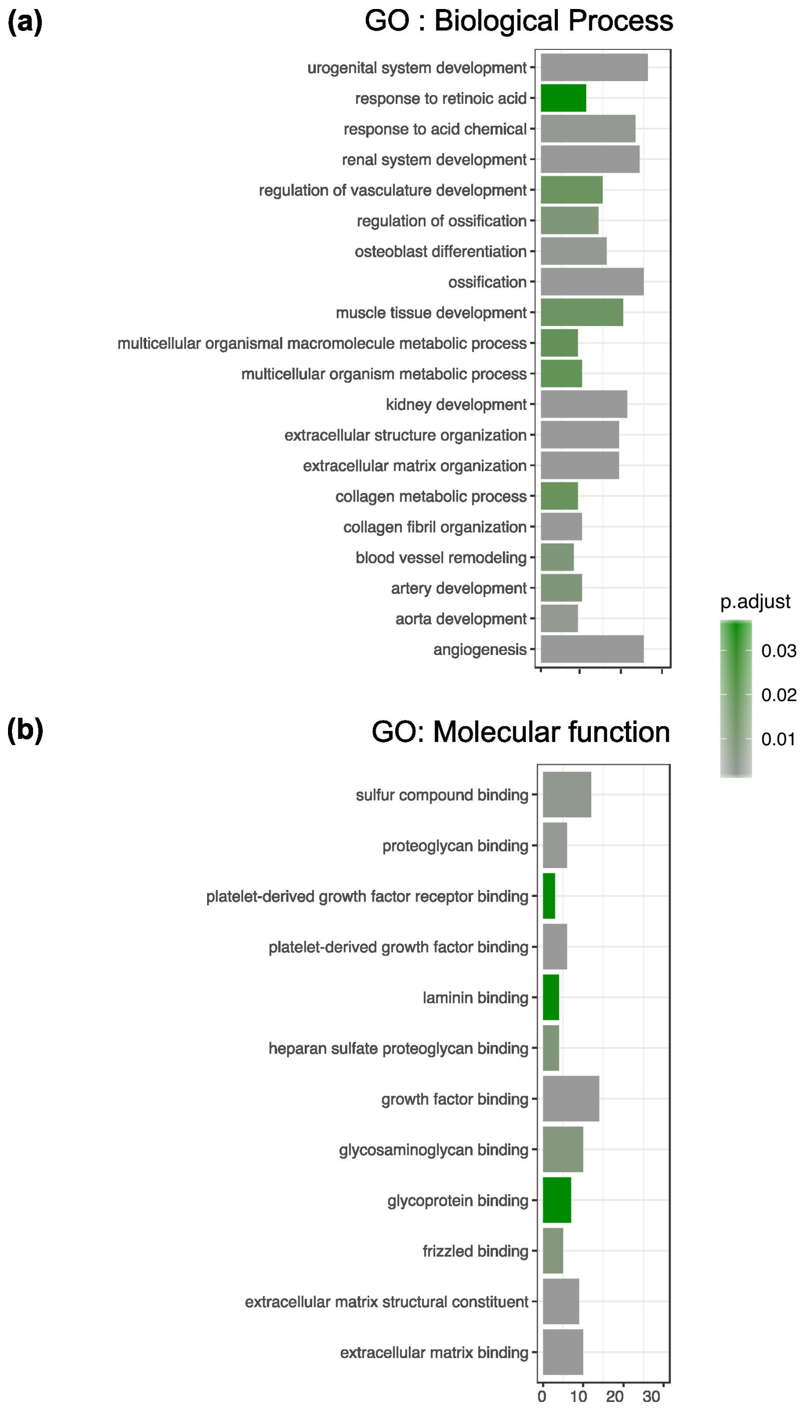

Supplementary Figure 9 Gene ontology analysis of SU enriched transcripts commonly identified by MAST and scDD in scRNA-seq. Histograms show the biological process **(a)** and molecular function **(b)** GO terms with an FDR adjusted *P*-value < 0.05. Shading of bar represents the exact p-adj. value.

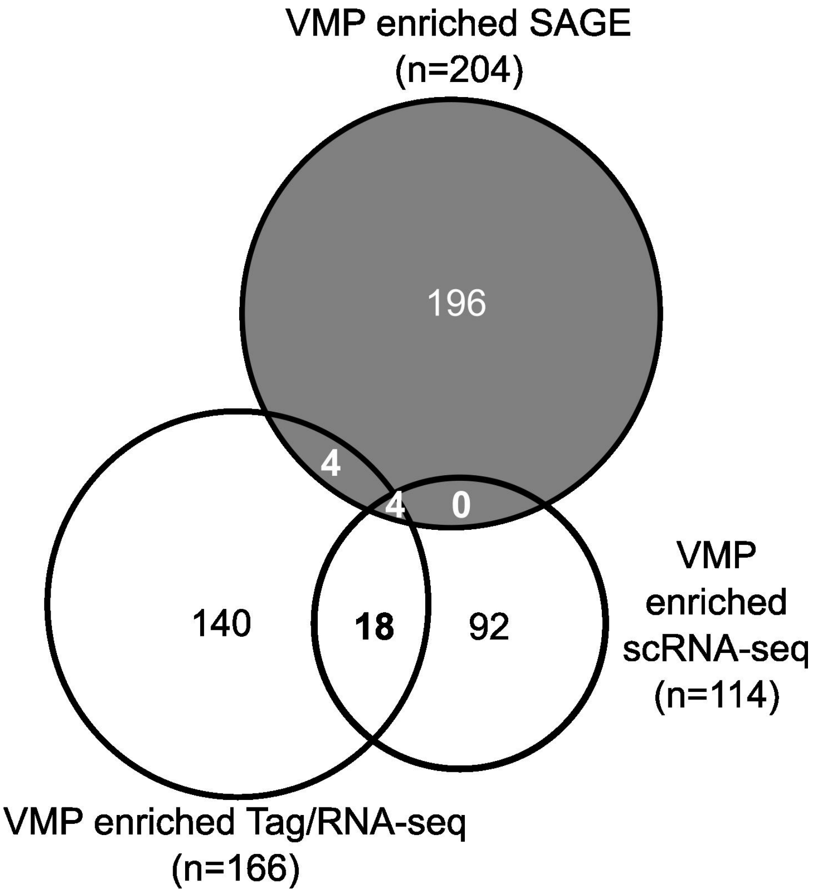

Supplementary Figure 10 Validation of differentially expressed VMP enriched transcripts with VMP enriched transcript identified by SAGE. VMP enriched transcripts previously identified by SAGE [4] were re-aligned to the Ensembl *Rnor_6.0* genome using BLAST [5] yielding 204 transcripts with characterised transcript IDs. A Venn diagram illustrates the overlap of VMP enriched differentially expressed transcripts identified by Tag-seq and RNA-seq with VMP enriched transcripts identified by SAGE. Among the transcripts co-identified between the current work and the previous SAGE study were Ptn and Dlk1; these have been experimentally validated as VMP-enriched using qrtPCR, in situ hybridisation and immunohistochemistry.

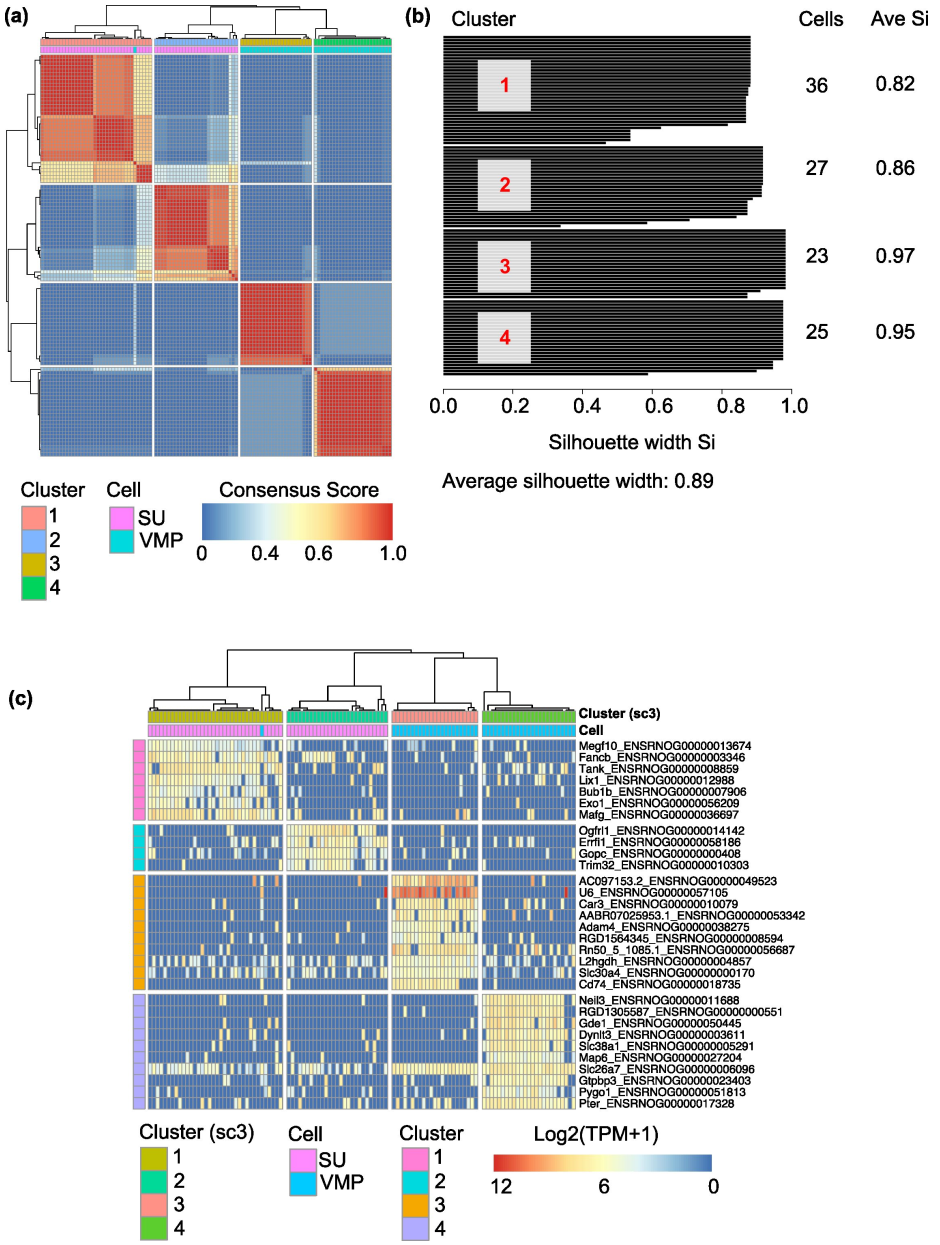

Supplementary Figure 11 Single cell subpopulation analysis using SC3. **(a)** Consensus clustering of VMP and SU cells using SC3 package identified 4 subpopulations (kmeans=4), two VMP and two SU subpopulations. Consensus matrix showing cluster stability. Blue indicates no consensus, and red indicates high consensus. **(b)** Silhouette plot for cluster identification. Silhouette plot showing the number of cells per cluster and the stability (Average width) of kmeans=4. The average width range between 0.82 and 0.97 indicates a strong cluster assignment. **(c)** Heatmap showing the expression levels of cluster marker genes for each of 4 clusters with AUC>0.75 and Holm adjusted *P*-value <0.05. Data represented as log2(TPM+1).

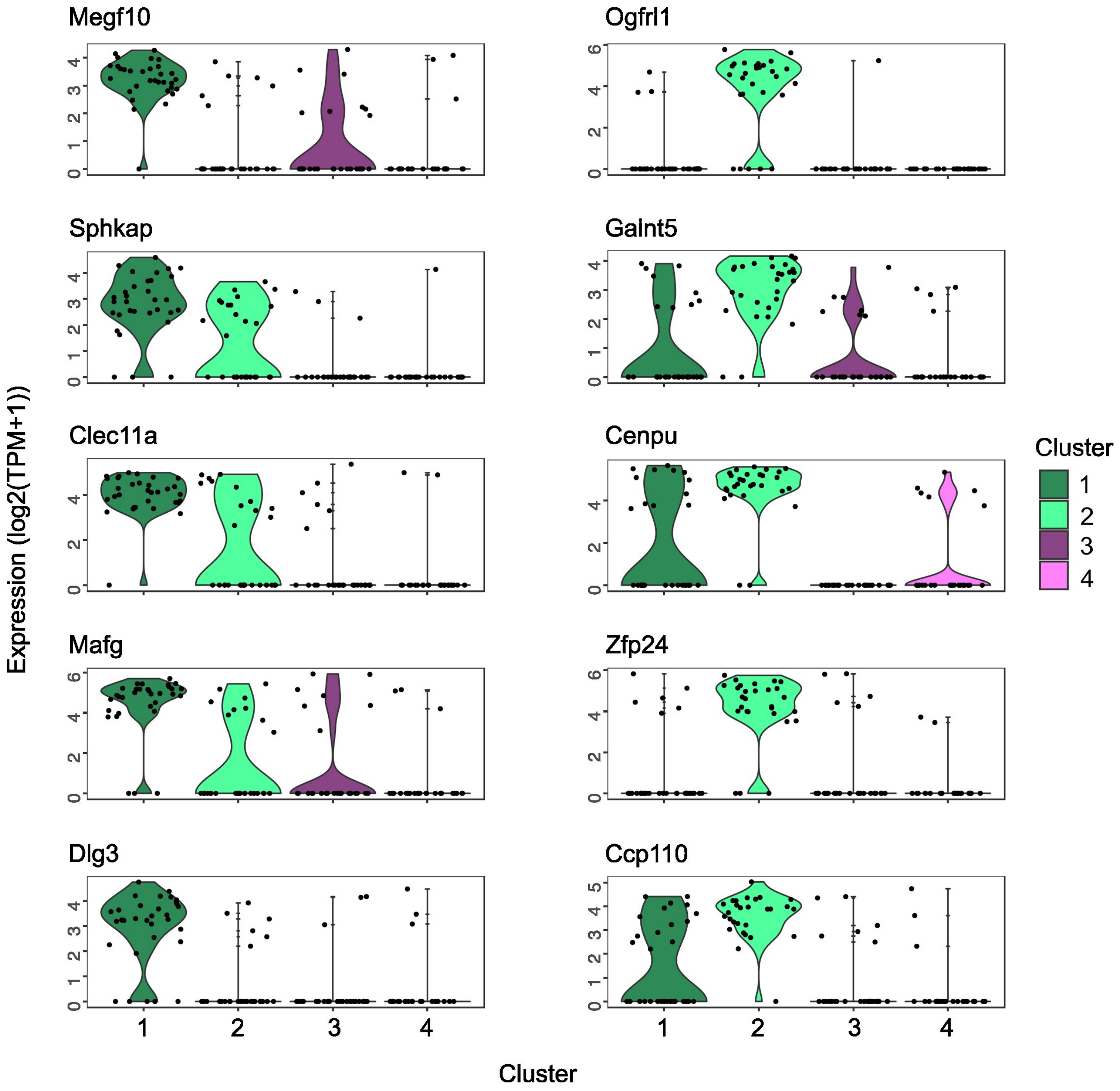
Megf10
Ogfrl1
Sphkap
Galnt5
Clec11a
Cenpu
Mafg
Zfp24
Dlg3
Ccp110
Expression (log2(TPM+1))
Cluster
1
2
3
4
Cluster

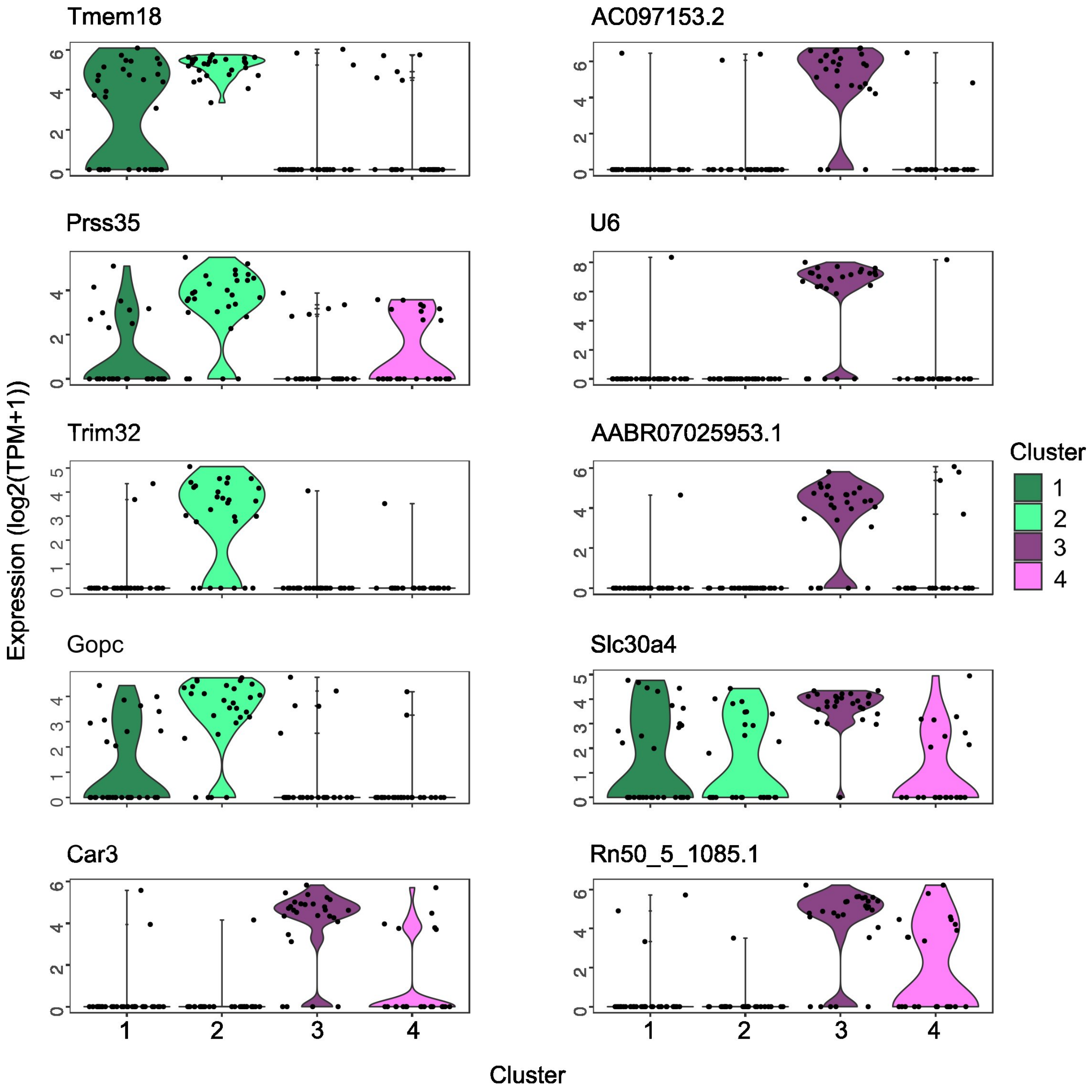
Tmem18
AC097153.2
Prss35
U6
Trim32
AABR07025953.1
Gopc
Slc30a4
Car3
Rn50_5_1085.1
Expression (log2(TPM+1))
Cluster
Cluster
1
2
3
4

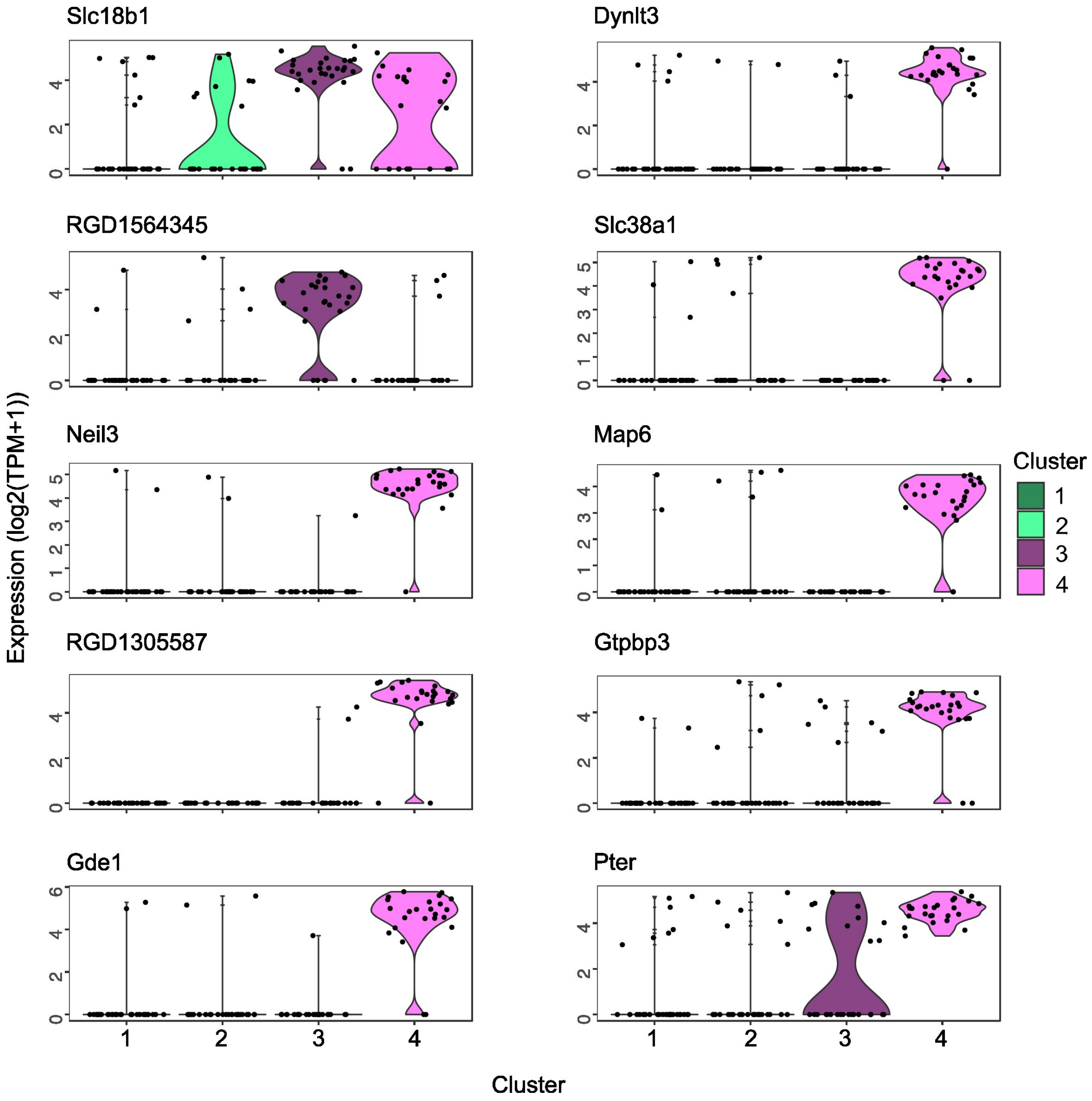
Slc18b1
Dynlt3
RGD1564345
Slc38a1
Neil3
Map6
RGD1305587
Gtpbp3
Gde1
Pter
Expression (log2(TPM+1))
Cluster
1
2
3
4

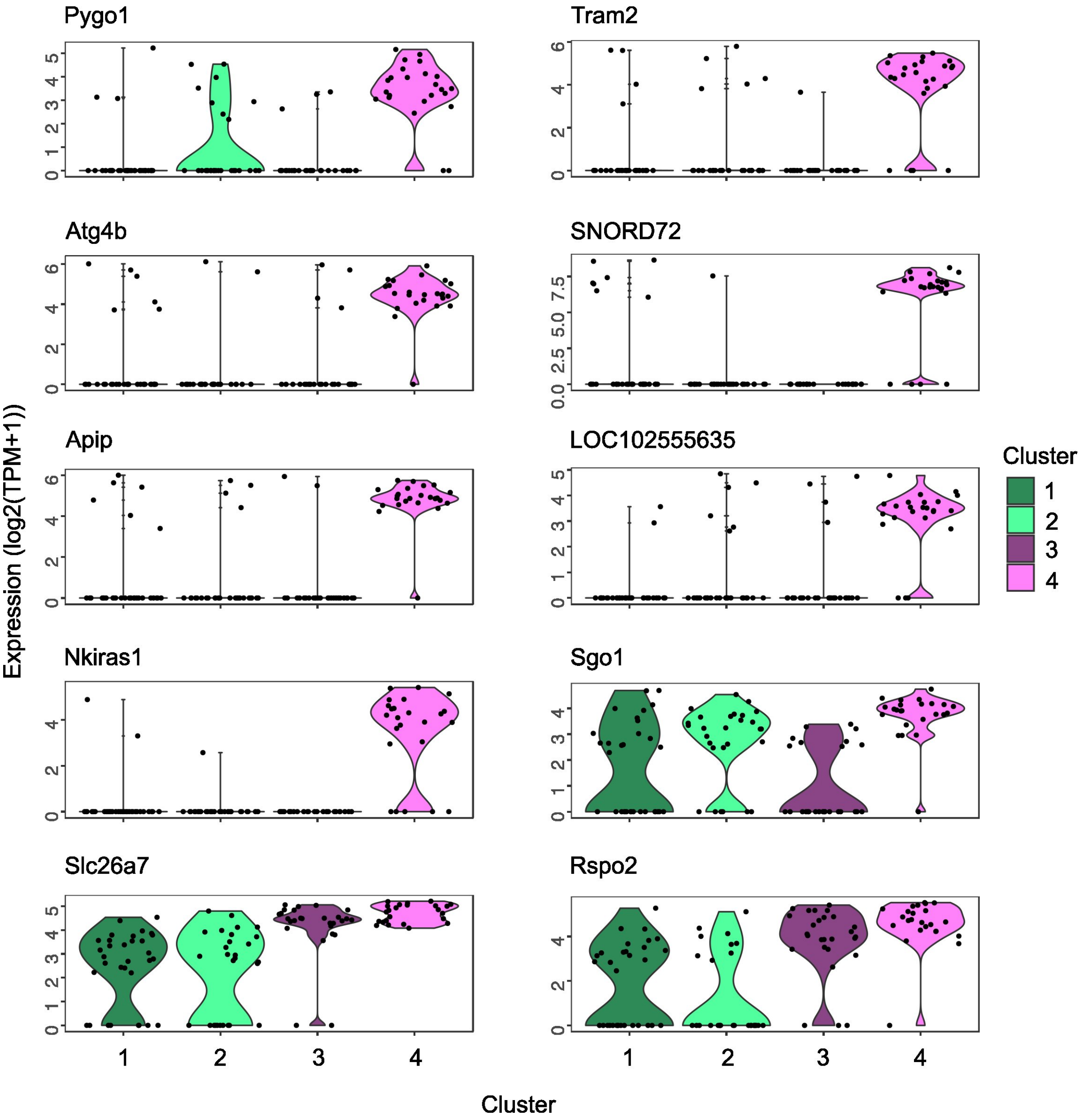

Supplementary Figure 12 Frequency distributions of expression of differentially expressed transcripts representing markers of the four cell clusters. Expression is presented as $\log_2$(TPM+1). Width of the violin plot indicates frequency of cells with that expression level.

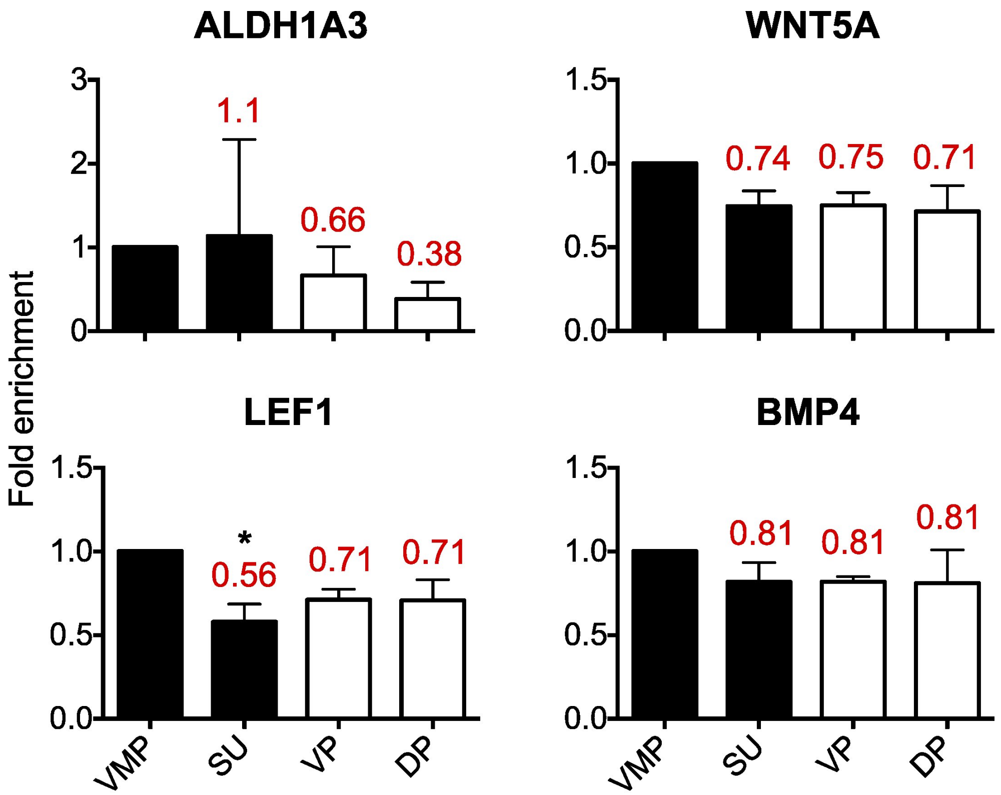

Supplementary Figure 13 Validation of VMP and SU candidate marker mRNA expression in female and male P0 rat urogenital sinus whole tissues. Quantitative real-time PCR of mRNA (qPCR) showed little difference in levels of known SU candidate markers between VMP and SU and between male tissues. Data is represented as mean fold difference to VMP ± SD (labeled in red) of duplicate biological replicates and duplicate technical replicates (n=4). Significance was detected using One-way ANOVA with TUKEY multiple comparison *p<0.05.

Supplementary References

1. Orr, B., et al., *Identification of stromally expressed molecules in the prostate by tag-profiling of cancer-associated fibroblasts, normal fibroblasts and fetal prostate.* Oncogene, 2012. **31**(9): p. 1130-42.
2. Nash, C., et al., *Genome-wide analysis of AR binding and comparison with transcript expression in primary human fetal prostate fibroblasts and cancer associated fibroblasts.* Mol Cell Endocrinol, 2017.
3. Macosko, E.Z., et al., *Highly Parallel Genome-wide Expression Profiling of Individual Cells Using Nanoliter Droplets.* Cell, 2015. **161**(5): p. 1202-1214.
4. Vanpoucke, G., et al., *Transcriptional profiling of inductive mesenchyme to identify molecules involved in prostate development and disease.* Genome Biol, 2007. **8**(10): p. R213.
5. Altschul, S.F., et al., *Basic local alignment search tool.* J Mol Biol, 1990. **215**(3): p. 403-10.
